# Revolutionizing Sun Protection: Emerging Nanotechnologies Shaping the Future of Sunscreens

**DOI:** 10.1021/acsptsci.5c00738

**Published:** 2026-03-03

**Authors:** Millena de S. Afonso, Pamella M. de Souza, Adriany L. dos Santos, Flávia A. do Carmo, Zaida M. F. de Freitas

**Affiliations:** † Faculty of Pharmacy, 28125 Federal University of Rio de Janeiro, Rio de Janeiro 21941-902, Brazil; ‡ Center for Microorganisms’ Investigation, Biomedical Institute, 28110 Federal Fluminense University, Niterói 24020-150, Brazil

**Keywords:** sunscreen, nanotechnology, nanosystems, efficacy, safety

## Abstract

Sun protection has become a significant public health issue in recent decades due to the high incidence of skin diseases related to excessive exposure to ultraviolet (UV) rays. The use of nanosystems in sunscreens represents a promising innovation in the pharmaceutical and cosmetic fields. This paper aims to map and characterize studies on the use of nanotechnology in sunscreens. This account identifies, categorizes, and describes the effectiveness and safety of these interventions. Searches were conducted in MEDLINE (via PubMed), Embase, Lilacs (BVS), Scopus, Web of Science, and Google Scholar. Fifty-four studies that described or evaluated the development of formulations associated with nanotechnology were included. The results suggest that nanotechnology can significantly enhance pharmaceutical sunscreen formulations by improving both the formulation performance and UV filter efficacy. However, further studies are needed, especially safety studies aimed at avoiding undesirable effects, such as systemic absorption. Therefore, the use of nanotechnology-based active delivery systems has become a widely adopted strategy for developing improved formulations.

## Graphical abstract

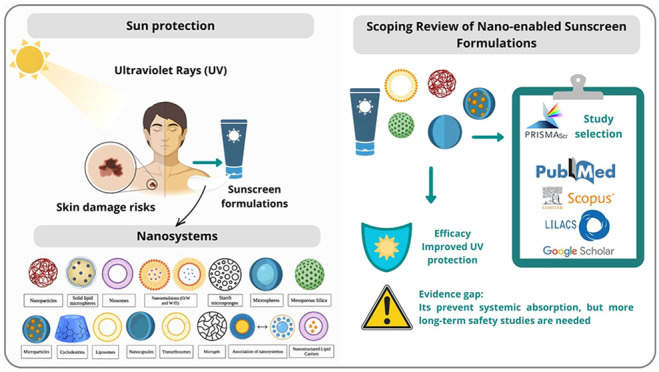

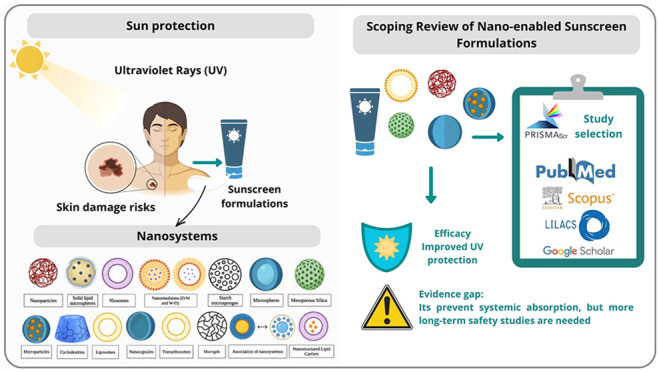

Sun protection has become a significant public health issue in recent decades due to the high incidence of skin diseases related to excessive exposure to ultraviolet (UV) rays.^1^ UV radiation is a well-established risk factor for several clinical conditions, including sunburn, photoaging, and skin cancers such as malignant melanoma and squamous cell carcinoma^2–4^

The use of sunscreens is widely recommended as an essential preventive measure against radiation-induced skin damage, as they contain chemical and/or physical filters capable of absorbing, reflecting, or dispersing UVA and UVB radiation.^5,6^ Regular use is associated with a decrease not only in the incidence of sunburn, but also in the risk of developing skin cancer.^7^ Therefore, sunscreen protects the skin immediately and complements long-term preventive strategies aimed at maintaining skin health.^8^

However, challenges related to the efficacy and safety of the active ingredients used in these products remain a constant concern.^7,9^ Nanosystems in sunscreens represent a promising innovation in the pharmaceutical and cosmetic fields, as they can improve photoprotective efficacy and product safety.^3,10^ The incorporation of filters into nanostructured systems allows for greater stability and solubility of the actives, more controlled release of sunscreens on the skin surface, and reduces irritation.^10^

Nanoparticles, liposomes, niosomes, and other nanoscale drug delivery systems are being tested for their ability to increase the photostability of the actives (a parameter that refers to the ability of the filter to maintain its functionality after exposure to UV radiation) and, specifically, reduce the penetration of sunscreens into the deeper layers of the skin, consequently minimizing potential toxic effects such as endocrine dysfunction and possible immunological reactions.^3,10–13^

Despite the potential benefits of nanosystems, there are significant gaps in the literature that need to be addressed. Their application is still surrounded by relevant challenges and controversies: the long-term safety and bioavailability of ingredients encapsulated in nanomaterials are not yet fully understood, with toxicological safety being one of the main points of debate related to the use of nanomaterials.^14^

In addition, the variability in evaluation methods and the lack of standardization among studies make it difficult to compare results and obtain definitive conclusions.^7,9^ Thus, although nanotechnology represents a promising strategy for improving photoprotective formulations, its application must be accompanied by a critical and cautious analysis of the potential risks, benefits, and existing knowledge gaps.

Therefore, a comprehensive review of the existing literature is crucial to map the available information and identify areas that require further research. The objective of this study was to conduct a scoping review of the application of nanotechnology in sunscreens, mapping the available scientific literature, categorizing the different types of formulations, and critically analyzing the evidence regarding their safety.

## Results

A total of 5703 references were identified. Of these, 1221 duplicate publications were automatically removed, and a total of 4482 records were included for initial screening. After reading the titles and abstracts, 4272 studies were excluded because they did not meet the established eligibility criteria: irrelevance to the topic, lack of application of nanosystems, and unrelated safety and experimental models. 210 were included for full-text review. A detailed analysis of these studies resulted in the inclusion of 54 studies for data synthesis. The study selection process is described in Figure 1.

**Figure 1:**
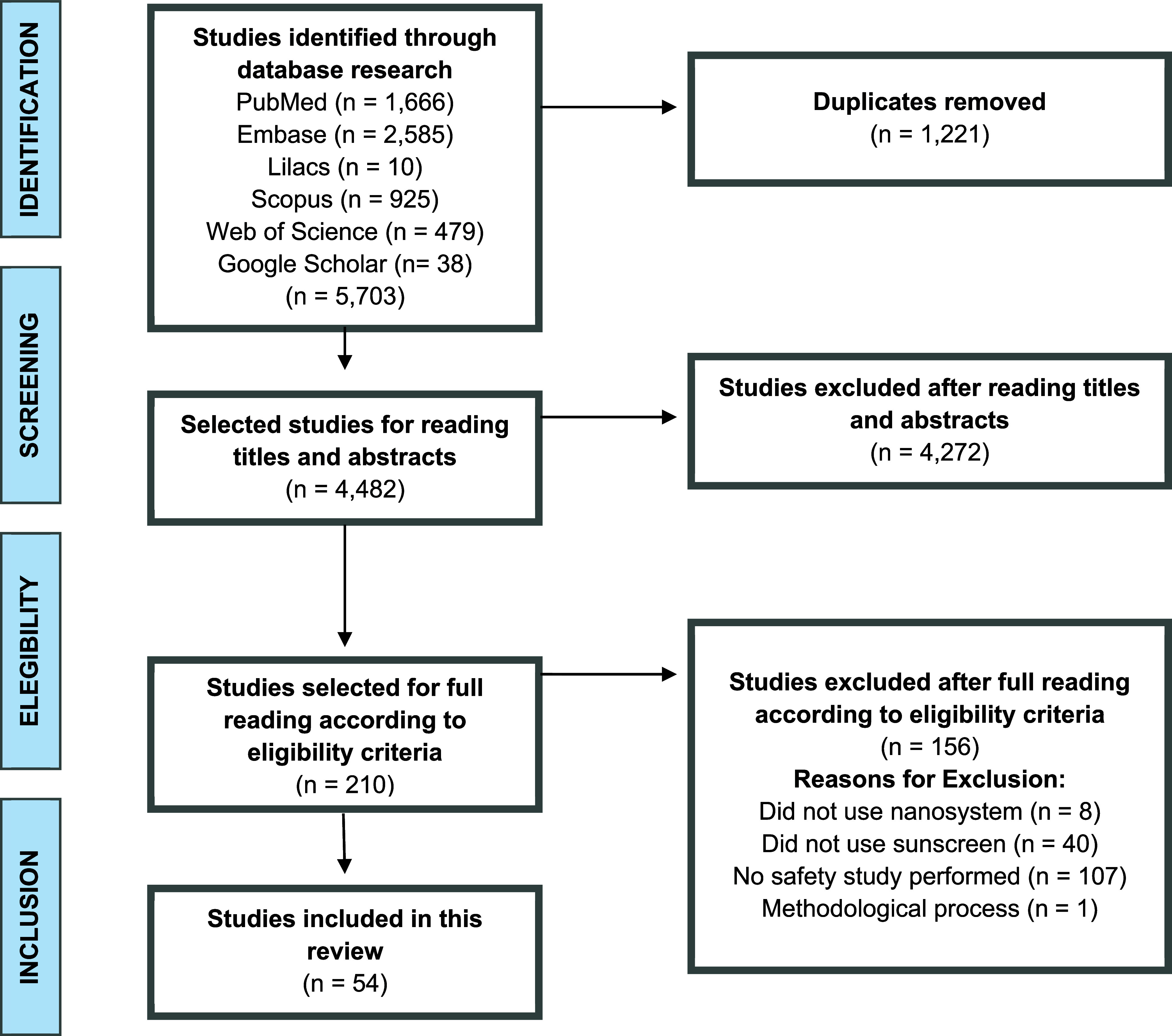
PRISMA flowchart describes the search results in the databases and the reference list of the selected studies.

Among the included studies (*n* = 54), all were primary studies, divided into experimental (*n* = 51) and clinical (*n* = 3) research. Fifteen were European studies, 19 were Asian, 15 were Brazilian, three were studies originating in Oceania, and two were North American (Figure 2).

**Figure 2:**
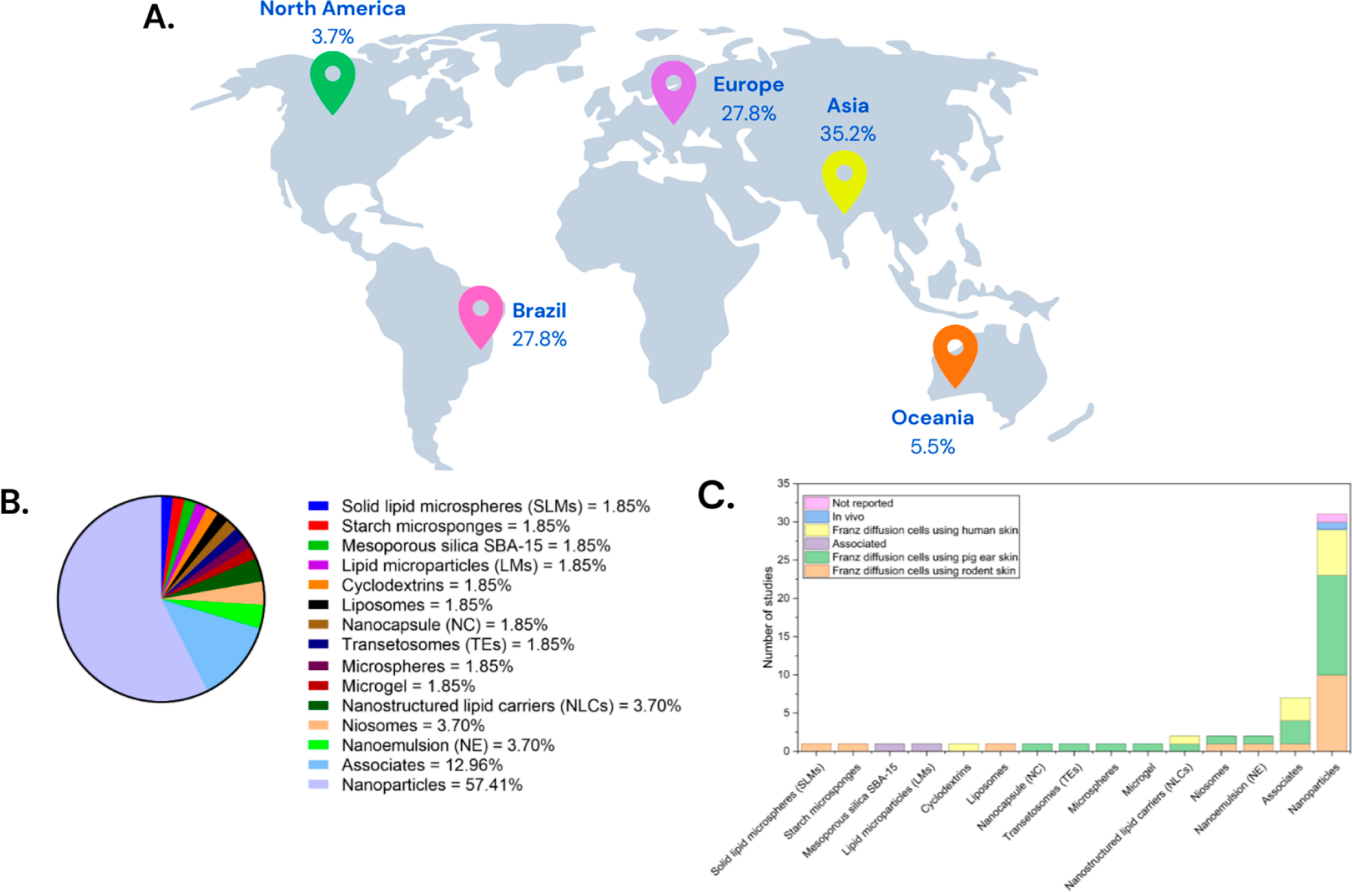
Quantitative parameters of the articles selected in the review: geographic distribution, types of systems used, and experimental safety models employed in the analyzed studies. (A) Percentage distribution of studies according to the geographic region of origin, with a predominance of research conducted in Asia (35.2%), Europe (27.8%), Brazil (27.8%), Oceania (5.5%), and North America (3.7%). (B) Relative frequency of the different types of nanostructured systems investigated. (C) Number of studies per system type, according to the experimental model employed, including *in vivo* assays, Franz diffusion using pig, rodent, and human skin, associated models, and unreported studies.

There is a predominance of studies conducted in Asia (35.2%), followed by Europe (27.8%) and Brazil (27.8%), while North America (3.7%) and Oceania (5.5%) show more limited participation (Figure 2A). This reflects not only the strong presence of Asian and European groups but also the relevant role of Brazil in this field. On the other hand, the low representation of North America may be related to more restrictive regulatory differences, which could impact the volume of published research.

It can be observed that the most frequently used nanosystem in the studies included in this review is nanoparticles, probably due to the greater availability of previous data that facilitate development, comparison of results, and experimental validation (Figure 2B). Other studies pointed to the association of nanosystems (12.96%), as well as the use of nanoemulsions (NE) (3.70%), niosomes (3.70%), and nanostructured lipid carriers (NLCs) (3.70%), while other systems, such as solids lipid microspheres (SLMs), Mesoporous Silica SBA-15SBA-15, liposomes, cyclodextrins, and polymeric nanocapsules, appear sporadically (≅1.85% each) (Figure 2B). This profile indicates that, although there is a diversity of nanostructured systems, only a portion of them have been explored more consistently, highlighting research gaps for less studied nanosystems.

Methodological analysis reveals a clear predominance of studies based on Franz diffusion cells, mainly using pig ear skin and, to a lesser extent, rodent and human skin, in addition to a significant number of studies in which the method was not clearly reported (Figure 2C). This finding reinforces a recurring limitation in the literature: the dependence on *ex vivo* and *in vitro* models, which, although useful, do not faithfully reproduce the physiological conditions of human skin *in vivo*. The scarcity of clinical studies and long-term evaluations limits the extrapolation of results, and this will be discussed in this review.

Among the main carriers found in this review, vesicular, lipid, polymeric, porous, and inclusion complexes stand out (Table 1).

**Table 1:** Characteristics of the Studies Are Included

author/year	title	study design	country	study design (methodology used)	nanosystem used
Wissing, S. A.; Muller, R. H.; 2002	Solid Lipid Nanoparticles as Carrier for Sunscreens: *In Vitro* Release and *In Vivo* Skin Penetration	primary—experimental research	Germany	comparison of two different formulations (solid lipid nanoparticle (SLN) and conventional emulsion) as carrier systems for oxybenzone (OXY) sunscreen	SLN
Yener, G.; Incegül, T.; Yener, N.; 2003	Importance of Using Solid Lipid Microspheres as Carriers for UV Filters on the Example Octyl Methoxycinnamate	primary—experimental research	Turkey	an attempt was made to prepare SLM for OMS; OMC was tested as a simple absorbent in microspheres, placed in various vehicles, and investigated and compared concerning OMC release, skin penetration, and photostability	SLM
Jiménez, M. M.; Pelletier, J.; Bobin, M. F.; Martini, M. C.; 2004	Influence of Encapsulation on the *In Vitro* Percutaneous Absorption of Octyl Methoxycinnamate	primary—experimental research	Spain	to investigate the influence of octyl methoxycinnamate poly(ε-caprolactone) nanocapsules (NC) on the *in vitro* percutaneous absorption of OMC compared to the same oil/water (O/W) and water/oil (W/O) emulsions	NC
Luppi, B.; Cerchiara, T.; Bigucci, F.; Basile, R.; Zecchi, V.; 2004	Polymeric Nanoparticles Composed of Fatty Acids and Polyvinyl Alcohol for topical Application of Sunscreens	primary—experimental research	Italy	modify polyvinyl alcohol 10,000 (PVA) hydrophobically with fatty acids (FAs) to obtain PVA-FA derivatives for the preparation of lipophilic polymeric nanoparticles capable of preventing the movement of benzophenone-3 (BZP) toward the skin	polymeric nanoparticles
Alvarez-Roman, R.; Naik, A.; Kalia, Y. N.; Guy, R. H.; Fessi, H.; 2004	Enhancement of Topical Delivery from Biodegradable Nanoparticles	primary—experimental research	France	to determine whether and how encapsulation of lipophilic compounds (such as the sunscreen OMC; Parsol MCX) in polymeric nanoparticles can improve topical application to the skin	polymeric nanoparticles
Simeoni, S.; Scalia, S.; Tursilli, R.; Benson, H.; 2006	Influence of Cyclodextrin Complexation on the *In Vitro* Human Skin Penetration and Retention of the Sunscreen Agent, Oxybenzone	primary—experimental research	Italy	to investigate the influence of cyclodextrins on the cutaneous availability of the sunscreen OXY; the interaction between OXY and the hydrophilic derivatives (hydroxypropyl-α-cyclodextrin (HP-α-CD), sulfobutyl ether-β-cyclodextrin (SBE-β-CD), and hydroxypropyl-cyclodextrin (HP-c-CD)) was studied in water by phase solubility analysis	cyclodextrins
Scalia, S.; Mezzena, M.; Iannuccelli, V.; 2007	Influence of Solid Lipid Microparticle Carriers on Skin Penetration of the Sunscreen Agent, 4-Methyl benzylidene Camphor	primary—experimental research	Italy	to prepare lipid microparticles (LMs) loaded with the sunscreen 4-methyl benzylidene camphor (4-MBC), to achieve reduced skin penetration of this UV filter	LMs
Anumansirikul, N.; Wittayasuporn, M.; Klinubol, P.; Tachaprutinun, A.; Wanichwecharungruang, S. P.; 2008	UV-Screening Chitosan Nanocontainers: Increasing the Photostability of Encapsulated Materials and Controlled Release	primary—experimental research	Thailand	the use of UV-absorbing polymeric carrier particles; chitosan derivatives with replacement of the UV-absorbing functionality were synthesized and prepared in carrier systems; the encapsulation of a model compound, photoisomerizable 2-ethylhexyl-4-methoxycinnamate (EHMC) was performed	polymeric nanoparticles
Wu, J.; Liu, W.; Xue, C.; Zhou, S.; Lan, F.; Bi, L.; Xu, H.; Yang, X.; Zeng, F. D.; 2009	Toxicity and Penetration of TiO_2_ Nanoparticles in Hairless Mice and Porcine Skin after Subchronic Dermal Exposure	primary—experimental research	China	to investigate the penetration and potential toxicity of titanium dioxide (TiO_2_) nanoparticles after their dermal exposure *in vitro* and *in vivo*	titanium dioxide nanoparticles (TiO_2_-NPs)
Weiss-Angeli, V.; Bourgeois, S.; Pelletier, J.; Guterres, S. S.; Fessi, H.; Bolzinger, M. A.; 2010	Development of an Original Method to Study Drug Release from Polymeric Nanocapsules in the Skin	primary—experimental research	Brazil	to investigate the distribution and release profile in the skin of a lipophilic molecule, octyl methoxycinnamate, loaded in NC of poly(ε-caprolactone) (OMC-NC)	poly(ε-caprolactone) NC
Senzui, M.; Tamura, T.; Miura, K.; Ikarashi, Y.; Watanabe, Y.; Fujii, M.; 2010	Study on Penetration of Titanium Dioxide (TiO_2_) Nanoparticles into Intact and Damaged Skin *In Vitro*	primary—experimental research	Japan	to focus on the skin penetration of TiO_2_ nanoparticles *in vitro* with different TiO_2_ dispersibility and skin conditions	TiO_2_-NPs
Vettor, M.; Bourgeois, S.; Fessi, H.; Pelletier, J.; Perugini, P.; Pavanetto, F.; Bolzinger, M. A.; 2010	Skin Absorption Studies of Octyl-methoxycinnamate Loaded Poly(d,l-lactide) Nanoparticles: Estimation of the UV Filter Distribution and Release Behavior in Skin Layers	primary—experimental research	Italy	to evaluate the percutaneous absorption of OMC released from PLGA polymeric nanoparticles loaded with OMC, formulated in an emulsion gel (OMC-NP emulgel) compared to a nonencapsulated OMC emulsion gel (OMC-emulgel)	PLA polymeric nanoparticles
Siqueira, N. M.; Contri, R. V.; Paese, K.; Beck, R. C. R.; Pohlmann, A. R.; Guterres, S. S.; 2011	Innovative Sunscreen Formulation Based on Benzophenone-3-Loaded Chitosan-Coated Polymeric Nanocapsules	primary—experimental research	Brazil	to evaluate the effect of cationic coating of polymeric NC in sunscreen formulations on the *in vitro* skin penetration of BZP	polymeric nanoparticles
Marcato, P. D.; Caverzan, J.; Rossi-Bergmann, B.; Pinto, E. F.; Machado, D.; Silva, R. A.; Justo, G. Z.; Ferreira, C. V.; Duran, N.; 2011	Nanostructured Polymer and Lipid Carriers for Sunscreen. Biological Effects and Skin Permeation	primary—experimental research	Brazil	to prepare and characterize poly(caprolactone) (PCL) and SLN to act as carriers BZP, aiming to improve the safety of sunscreens by increasing the sun protection factor (SPF), decreasing BZP penetration into the skin, and reducing the concentration of BZP in the sunscreen formulation	PPCL and SLN
Monteiro-Riviere, N. A.; Wiench, K.; Landsiedel, R.; Schulte, S.; Inman, A. O.; Riviere, J. E.; 2011	Safety Evaluation of Sunscreen Formulations Containing Titanium Dioxide and Zinc Oxide Nanoparticles in UVB Sunburned Skin: An *In Vitro* and *In Vivo* Study	primary—experimental research	United States	to evaluate the absorption and penetration of commercially available TiO_2_ and zinc oxide (ZnO) nanoparticles in sunscreen formulations in ultraviolet B (UVB)-damaged skin, *in vitro* and *in vivo*	TiO_2_ ZnO nanoparticles
Monteiro, M. S. D. D.; Ozzetti, R. A.; Vergnanini, A. L.; de Brito-Gitirana, L.; Volpato, N. M.; de Freitas, Z. M. F.; Ricci, E.; dos Santos, E. P.; 2012	Evaluation of Octyl *p*-Methoxycinnamate included in liposomes and cyclodextrins in antisolar preparations: preparations, characterizations and *in vitro* penetration studies	primary—experimental research	Brazil	to develop a new formulation containing OMC loaded in liposomes and cyclodextrins (CD) and compare several parameters in the different dispersions	liposomes and CD
Jirova, D.; Kejlova, K.; Pikal, P.; Kasparova, L.; Safarova, K.; Kovarikova, L.; Bendová, H.; Zalabak, D. E.; 2012	Effect of TiO_2_ Nanoparticle Size on Possible Skin Penetration *In Vitro*	primary—experimental research	Czech Republic	develop and modify titanium dioxide nanoparticles (TiO_2_-NPs) designed for specific applications: UV filters, antimicrobial functional textiles, or photoactive self-cleaning coating materials	TiO_2_-NPs
Hanno, I.; Anselmi, C.; Bouchemal, K.; 2012	Polyamide Nanocapsules and Nanoemulsions Containing Parsol (R) MCX and Parsol (R) 1789: *In Vitro* Release, *Ex Vivo* Skin Penetration and Photostability Studies	primary—experimental research	France	Parsol MCX and Parsol 1789 filters will be encapsulated, alone or mixed, in NCs in combination with ÿ-tocopherol and the results will be compared with the NE prepared under the same conditions as the NCs, but without monomers	NCs and NE
Gulson, B.; Wong, H.; Korsch, M.; Gomez, L.; Casey, P.; McCall, M.; McCulloch, M.; Trotter, J.; Stauber, J.; Green Oak, G.; 2012	Comparison of Dermal Absorption of Zinc from Different Sunscreen Formulations and Differing UV Exposure Based on Stable Isotope Tracing	Primary—Clinical research	Australia	the results of the pilot study were compared with those of the outdoor test that employed a formulation; a generic oil–water sunscreen formulation containing ZnO nanoparticles enriched in the stable isotope ^68^Zn tracer was the basis of these tests with UV exposure	ZnPs
Teixeira, Z; Dreiss, C. A.; Lawrence, M. J.; Heenan, R. K.; Machado, D.; Justo, G. Z.; Guterres, S. S.; Durán, N.; 2012	Retinyl Palmitate Polymeric Nanocapsules as Carriers of Bioactives	primary—experimental research	Brazil	to bring a better structural understanding of similar NCs comprising retinyl palmitate (RP), poly(d,l-lactide) (PLA) and surfactants (either Span 60 and Tween 80 or Pluronic 68) compared to equivalent nanospheres (without RP) and NE (without PLA but with surfactants)	NCs, nanospheres (NS) and NE
Miquel-Jeanjean, C.; Crepel, F.; Raufast, V.; Payre, B.; Datas, L.; Bessou-Touya, S.; Duplan, H.; 2012	Penetration Study of Formulated Nanosized Titanium Dioxide in Models of Damaged and Sun-Irradiated Skins	primary—experimental research	France	to evaluate *in vitro* the skin penetration of NPs-TiO_2_, included in sunscreen, in intact, damaged, irradiated, and irradiated damaged pig skin	TiO_2_-NPs
Mota, A. D. V.; de Freitas, Z. M. F.; Ricci, E.; Dellamora-Ortiz, G. M.; Santos-Oliveira, R.; Ozzetti, R. A.; Vergnanini, A. L.; Ribeiro, V. L.; Silva, R. S.; dos Santos, E. P.; 2013	*In Vivo* and *In Vitro* Evaluation of Octyl Methoxycinnamate Liposomes	primary—experimental research	Brazil	to develop and evaluate a liposomal nanosystem (liposome/OMC) of OMC to obtain a sunscreen formulation with greater safety and efficacy by retaining the OMC for longer in the stratum corneum	liposome
Puglia, C.; Damiani, E.; Offerta, A.; Rizza, L.; Tirendi, G. G.; Tarico, M. S.; Curreri, S.; Bonina, F.; Perrotta, R. E.; 2014	Evaluation of Nanostructured Lipid Carriers and Nanoemulsions as Carriers for UV filters: characterization, *In Vitro* Penetration and Photostability Studies	primary—experimental research	Italy	NLC and NE were formulated to optimize the topical application of different and widespread UVA or UVB sunscreens (ethylhexyl triazine (EHT), diethylamino hydroxybenzoyl hexyl benzoate (DHHB), bemotrizinol (Tinosorb S), octyl methoxy)	NLC and NE
Shetty, P. K.; Venuvanka, V.; Jagani, H. V.; Chethan, G. H.; Ligade, V. S.; Musmade, P. B.; Nayak, U. Y.; Reddy, M. S.; Kalthur, G.; Udupa, N.; Rao, C. M.; Mutalik, S.; 2015	Development and Evaluation of Sunscreen Creams Containing Morin-Encapsulated Nanoparticles for Enhanced UV Radiation Protection and Antioxidant Activity	primary—experimental research	India	to develop new sunscreen creams containing polymeric nanoparticles (NPs) of ZnO and TiO_2_ to improve morin penetration into the skin, with minimal absorption into the systemic circulation	NPs
Crosera, M.; Prodi, A.; Mauro, M.; Pelin, M.; Florio, C.; Bellomo, F.; Adami, G.; Apostoli, P.; De Palma, G.; Bovenzi, M.; Campanini M.; Filon F. L.; 2015	Titanium Dioxide Nanoparticle Penetration into the Skin and Effects on HaCaT Cells	primary—experimental research	Italy	to study the *in vitro* cutaneous absorption of TiO_2_-NPs in intact and damaged human skin	TiO_2_-NPs
Cerqueira-Coutinho, C.; Santos-Oliveira, R.; dos Santos, E.; Mansur, C. R.; 2015	Development of a Photoprotective and Antioxidant Nanoemulsion Containing Chitosan as an Agent for Improving Skin Retention	primary—experimental research	Brazil	to develop a photoprotective and antioxidant oil-in-water NE containing chitosan and the organic sunscreens BZP, diethylamino hydroxybenzoyl hexyl benzoate, octocrylene, and octyl methoxycinnamate, as well as antioxidant pomegranate extract	NE
Xie, G.; Lu, W.; Lu, D.; 2015	Penetration of Titanium Dioxide Nanoparticles through Slightly Damaged Skin *In Vitro* and *In Vivo*	primary—experimental research	China	to investigate the penetration of TiO_2_-NPs through slightly injured and intact skin *in vitro* and *in vivo*	TiO_2_-NPs
Cerqueira-Coutinho, C. S.; De Campo, V. E. B.; Rossi, A L.; Veiga, V. F.; Holandino, C.; Freitas, Z. M. F.; Ricci-Junior, E.; Mansur, C. R. E.; Santos, E. P.; Santos-Oliveira, R.; 2016	Comparing *In Vivo* Biodistribution with Radiolabeling and Franz Cell Permeation Assay to Validate the Efficacy of Both Methodologies in the Evaluation of Nanoemulsions: A Safety Approach	primary—experimental research	Brazil	to develop, characterize, and evaluate a NE containing OMC, comparing the safety of the formulation	NE
de Oliveira, C. A.; Dario, M. F.; Sarruf, F. D.; Mariz, I. F. A.; Velasco, M. V. R.; Rosado, C.; Baby, A. R.; 2016	Safety and Efficacy Evaluation of Gelatin-Based Nanoparticles Associated with UV filters	primary—experimental research	Brazil	to investigate the preclinical safety of gelatin nanoparticles (GNPs) using an *in vitro* method and to evaluate the clinical safety and efficacy of combining GNPs with three commonly used chemical UV filters (ethylhexyl dimethyl PABA, ethylhexyl methoxycinnamate, and methoxydibenzoylmethane)	GNPs
Gilbert, E.; Roussel, L.; Serre, C.; Sandouk, R.; Salmon, D.; Kirilov, P.; Haftek, M.; Falson, F.; Pirot, F.; 2016	Percutaneous Absorption of Benzophenone-3 Loaded Lipid Nanoparticles and Polymeric Nanocapsules: A Comparative Study	primary—experimental research	France	to compare the percutaneous absorption and cutaneous bioavailability of BZP loaded in SLN, NLC, nanostructured polymeric lipid carriers (NPLC) and NC	SLN, NLC, NPLC and NC
Arslan Azizoglu, G.; Tuncay Tanriverdi, S.; Aydin Kose, F.; Ballar Kirmizibayrak, P.; Ozer, O.; 2017	Dual-prevention for UV-induced skin damage: incorporation of melatonin-loaded elastic niosomes into octyl methoxycinnamate pickering emulsions	primary—experimental research	Turkey	melatonin-loaded elastic niosomes (MEL) and octyl OMC Pickering emulsion were prepared separately, aiming to maintain the accumulation of OMC in the outer layers of the skin, while MEL-loaded elastic niosomes can penetrate the deeper layers of the skin	niosomes
Joshi, H.; Hegde, A. R.; Shetty, P. K.; Gollavilli, H.; Managuli, R. S.; Kalthur, G.; Mutalik, S.; 2018	Sunscreen Creams Containing Naringenin Nanoparticles: Formulation Development and *In Vitro* and *In Vivo* Evaluations	primary—experimental research	India	to develop sunscreen creams containing naringenin NPs for photoprotective effects	NPs
Andreo, N.; Bim, A. V. K.; Kaneko, T. M.; Kitice, N. A.; Haridass, I. N.; Abd, E.; Lopes, P. S.; Thakur, S. S.; Parekh, H. S.; Roberts, M. S.; Grice, J. E.; Benson, H. A. E.; Leite-Silva, V. R.; 2018	Development and Evaluation of Lipid Nanoparticles Containing Natural Botanical Oil for Sun Protection: Characterization and *In Vitro* and *In Vivo* Human Skin Permeation and Toxicity	primary—experimental research	Brazil	develop solid colloidal nanocarriers composed of a lipid base and vegetable oils, incorporating the UV filter octyl OMC to achieve a high SPF with a reduced concentration of chemical UV filters	solid colloidal nanocarriers
Viswanathan, K.; Vaiyamalai, R.; Bharathi Babu, D.; Mala Priyadharshini, M. L.; Raman, M.; Dhinakarraj, G.; 2018	Ketoconazole-Conjugated ZnO Nanoparticles Based Semisolid Formulation and Study Their Impacts on Skin Disease	primary—clinical research	India	ZnO NPs conjugated with ketoconazole to evaluate their impacts on skin diseases	ZnO NPs
Cerqueira, C.; Nigro, F.; Campos, V. E. B.; Rossi, A.; Santos-Oliveira, R.; Cardoso, V.; Vermelho, A. B.; dos Santos, E. P.; Mansur, C. R. E.; 2019	Nanovesicle-Based Formulations for Photoprotection: A Safety and Efficacy Approach	primary—experimental research	Brazil	to prepare and characterize nanosystems formed by niosomes to be applied as organic sunscreens	niosomes
Bhuptani, R. S.; Patravale, V. B.; 2019	Starch Microsponges For Enhanced Retention and Efficacy of Topical Sunscreen	primary—experimental research	India	a formulation was developed based on starch microsponges as a key vehicle encapsulating BZ3	starch microsponges
Daré, R. G.; Costa, A.; Nakamura, C. V.; Truiti, M. C. T.; Ximenes, V. F.; Lautenschlager, S. O. S.; Sarmento, B.; 2020	Evaluation of Lipid Nanoparticles for Topical Delivery of Protocatechuic Acid and Ethyl Protocatechuate as a New Photoprotection Strategy	primary—experimental research	Brazil	to develop SLNs and NLCs for topical delivery of protocatechuic acid (P0) or ethyl protocatechuate (P2) as a photoprotection strategy	SLN
Rodrigues, L. R.; Jose, J.; 2020	Exploring the Photo Protective Potential of Solid Lipid Nanoparticle-Based Sunscreen Cream Containing Aloe vera	primary—experimental research	India	to formulate and evaluate six sunscreen creams in SLN, loaded with *Aloe vera* and determine their photoprotective potential	SLN
Holmes, A. M.; Kempson, I.; Turnbull, T.; Paterson, D.; Roberts, M. S.; 2020	Penetration of Zinc into Human Skin after Topical Application of Nano Zinc Oxide Used in Commercial Sunscreen Formulations	primary—experimental research	Australia	address the concern that zinc dissolution from ZnO NPs may penetrate human skin under various conditions: in the presence of sweat or if the skin barrier is impaired	ZnO NPs
Khabir, Z.; Holmes, A. M.; Lai, Y.-J.; Liang, L.; Deva, A.; Polikarpov, M. A.; Roberts, M. S.; Zvyagin, A. V.; 2021	Human Epidermal Zinc Concentrations after Topical Application of ZnO Nanoparticles in Sunscreens	primary—experimental research	Australia	quantifying the relative concentrations of endogenous and exogenous Zn using a rare stable isotope of zinc-67 and ZnO-NP sunscreen applied to excised human skin	ZnO NPs
Tomer, S.; Suh, H.; Zhou, A. G.; Yu, B.; Lewis, J.; Saltzman, M.; Girardi, M.; 2021	504 Nanoparticle Encapsulation Enhances Stability and Efficacy of Sunscreen Actives	primary—experimental research	United States	to verify the stability and efficacy of active sunscreens, free and encapsulated in biodegradable bioadhesive nanoparticles (BNP) exposed to UVR from a solar simulator	BNP
Daneluti, A. L. M.; Guerra, L. O.; Velasco, M. V. R.; do Rosário Matos, J.; Baby, A. R.; Kalia, Y. N.; 2021	Preclinical and Clinical Studies to Evaluate Cutaneous Biodistribution, Safety and Efficacy of UV Filters Encapsulated in Mesoporous Silica SBA-15	primary—clinical research	Brazil	to determine the cutaneous biodistribution of avobenzone (AVO), OXY and OMC incorporated into SBA-15 mesoporous silica, however, a previous publication by the group demonstrated that the delivery of OMC from a stick with nonincorporated filters and a stick with incorporated filters were not significantly different, so it was decided to quantify only OXY and AVO in these skin application studies	mesoporous silica SBA-15
Basto, R.; Andrade, R.; Nunes, C.; Lima, S. A. C.; Reis, S.; 2021	Topical Delivery of Niacinamide to Skin Using Hybrid Nanogels Enhances Photoprotection Effect	primary—experimental research	Portugal	hybrid nanogel was designed using carrageenan and poly(vinylpyrrolidone) polymers combined with jojoba oil as a permeation enhancer; three different types of transethosomes were prepared by the thin film hydration method, differentiated by the presence of an edge activator or a permeation enhancer, to allow a controlled delivery of niacinamide (NIA)	transethosomes (TEs)
Kaur, J.; Anwer, M. K.; Sartaj, A.; Panda, B. P.; Ali, A.; Zafar, A.; Kumar, V.; Gilani, S. J.; Kala, C.; Taleuzzaman, M.; 2022	ZnO Nanoparticles of *Rubia cordifolia* Extract Formulation Developed and Optimized with QbD Application, Considering *Ex Vivo* Skin Permeation, Antimicrobial and Antioxidant Properties	primary—experimental research	India	to develop ZnO-Manjistha nanoparticles extract (ZnO-MJE) and investigate its transdermal delivery as well as antimicrobial and antioxidant activity	NP
Ghazwani, M.; Hani, U.; Alqarni, M. H.; Alam, A.; 2023	Development and Characterization of Methyl-anthranilate-Loaded Silver Nanoparticles: A Phytocosmetic Sunscreen Gel for UV Protection	primary—experimental research	Saudi Arabia	develop a UV-protective sunscreen gel using silver nanoparticles loaded with methyl anthranilate (MA) (MA-AgNPs)	AgNPs
Ma, Q.; Zhang, Y.; Huangfu, Y.; Gao, S.; Zhou, C.; Rong, H.; Deng, L.; Dong, A.; Zhang, J.; 2023	Solid SiO_2_-Sealed Mesoporous Silica for Synergistically Combined Use of Inorganic and Organic Filters to Achieve Safe and Effective Skin Protection from All-Band UV Radiation	primary—experimental research	China	entrap mesoporous silica nanoparticles (MSN) in TiO_2_ nanocrystals (MSNTiO_2_) and MSN in DHHB (MSN-DHHB), sealed by a silica layer, using the sol–gel method using tetraethyl orthosilicate (TEOS)	MSN
Sousa, I. P.; Landim, A. C. T.; Ribeiro, B. C. C.; Cintra, E. R.; Silva, L. M.; Nascimento, T. L.; Lima, E. M.; Silva, L. A. D.; Diniz, D. G. A.; 2023	Improved Photostability and Skin Retention of Avobenzone Encapsulated in Compatible Nanostructured Lipid Carriers	primary—experimental research	Brazil	develop a NLC formulation encapsulating AVO, and evaluate its photostability to UV radiation and its ability to reduce AVO skin penetration, favoring its retention on the skin surface	NLCs
de Araújo, M. M.; Schneid, A. C.; Oliveira, M. S.; Mussi, S. V.; de Freitas, M. N.; Carvalho, F. C.; Bernes Junior, E. A.; Faro, R.; Azevedo, H.; 2024	NLC-Based Sunscreen Formulations with Optimized Proportion of Encapsulated and Free Filters Exhibit Enhanced UVA and UVB Photoprotection	primary—experimental research	Brazil	develop innovative sunscreen formulations using NLCs (SC-NLC) based on bacuri butter and raspberry seed oil	NLCs
Wang, W.; He, Q.-T.; Chen, Y.-F.; Wang, B.-H.; Xu, W.-Y.; Liu, Q.-L.; Liu, H.-M; 2024	Anti-UV Microgel Based on Interfacial Polymerization to Decrease Skin Irritation of High Permeability UV Absorber Ethylhexyl Methoxycinnamate	primary—experimental research	China	develop a carrier Microgel, consisting of poly(ethylene glycol dimethacrylate) (pEDGMA), synthesized using interfacial polymerization to reduce irritation and penetration of ethylhexyl methoxycinnamate (EHMC)	microgel
Zhang, J.; Zhang, S.; Yan, C.; Bi, J.; Han, X.; Liu, H.; 2024	Tint-Adjustable Pickering Emulsion Sunscreen Based on Polydopamine-Coated Silica Nanoparticles	primary—experimental research	China	develop a series of amorphous polydopamine-coated silica nanoparticles (SiO_2_-PDA) synthesized by a simple one-pot method	SiO_2_-PDA
Tarantini, A.; Jamet-Anselme, E.; Lam, S.; Haute, V.; Suhard, D.; Valle, N.; Chamel-Mossuz, V.; Bouvier-Capely, C.; Phan, G.; 2024	*Ex Vivo* Skin Diffusion And Decontamination Studies of Titanium Dioxide Nanoparticles	primary—experimental research	France	adapt an experimental model based on Franz diffusion cells and porcine skin explants to characterize the diffusion of TiO_2_ NPs in healthy and excised skin	TiO_2_ NPs
Shoaib Khan, H. M.; Butt, H.; Sohail, M.; Rehman, S.; Ramzan, N.; Abbas Malik, H. M.; 2025	Pharmaceutical Hybrid Nanogel of Nanoflavonoid and Zinc Oxide for Dermatological Applications	primary—experimental research	Pakistan	develop an effective nanogel formulation of nanoflavonoids and zinc oxide for the treatment and protection of various skin diseases	mesoporous silica nanoparticles
Gui, H.; Liu, H.; Cai, Y.; Nian, J.; Liu, L.; Song, Y.; Kye, S.; Zuo, S.; Yao, C.; 2025	Hyaluronic Acid-Grafted Titanium Dioxide Nanoparticles for Moisture-Retentive and Noncytotoxic Sunscreen Creams	primary—experimental research	Chine	develop NPs sequentially coated with aluminum hydroxide (Al(OH)_3_) and chemically grafted with hyaluronic acid (HA)	TiO_2_-NPs
Li, Z.; Chen, L.; Qiu, X.; 2025	Green Encapsulation of Avobenzone in Lignin Microspheres: A Promising Approach for Enhanced UV Protection and Photostability	primary—experimental research	Chine	develop lignin microspheres for encapsulation of AVO	lignin microspheres

In vesicular systems, liposomes are nanosystems formed by phospholipids organized in bilayers, allowing the encapsulation of hydrophilic and lipophilic compounds.^3^ Alternatively, niosomes use surfactants instead of phospholipids, overcoming limitations such as chemical instability and high cost.^16^ Transethosomes, derived from ethosomes and transferosomes, exhibit high deformability, which facilitates skin penetration and targeted release.^17^

Among the lipid systems, NEs, lipid microparticles, SLNs, and NCs stand out. NEs are oil/water or water/oil dispersions composed of an oil phase, an aqueous phase, and surfactants, the choice of which influences stability and biological interaction.^5,18^ Microparticles have a solid lipid matrix stabilized by surfactants, ensuring skin substantivity and low systemic absorption.^19^ SLNs act as physical filters and, combined with molecular filters, promote a synergistic effect in photoprotection.^1^ NCs, in turn, provide prolonged release of active ingredients, keeping them on the skin surface for longer, which is advantageous in UV blockers.^20^

In polymeric systems, microgels reduce skin irritation and control the penetration of active ingredients,^21^ while microsponges, consisting of porous microspheres with a high surface area, allow slow and controlled release, without significant permeation.^12^

In the porous systems group, mesoporous silica SBA-1 has an ordered pore structure, favoring the encapsulation and protection of sunscreens against degradation.^8^

Finally, in inclusion complexes, cyclodextrins trap hydrophobic compounds within their cavities, increasing solubility and modulating percutaneous absorption according to the desired application.^22^ Figure 3 presents an illustrative image of the particulate and vesicular lipid nanosystems, the safety methodology used for their evaluation, and the skin types employed in the tests described in this article.

**Figure 3:**
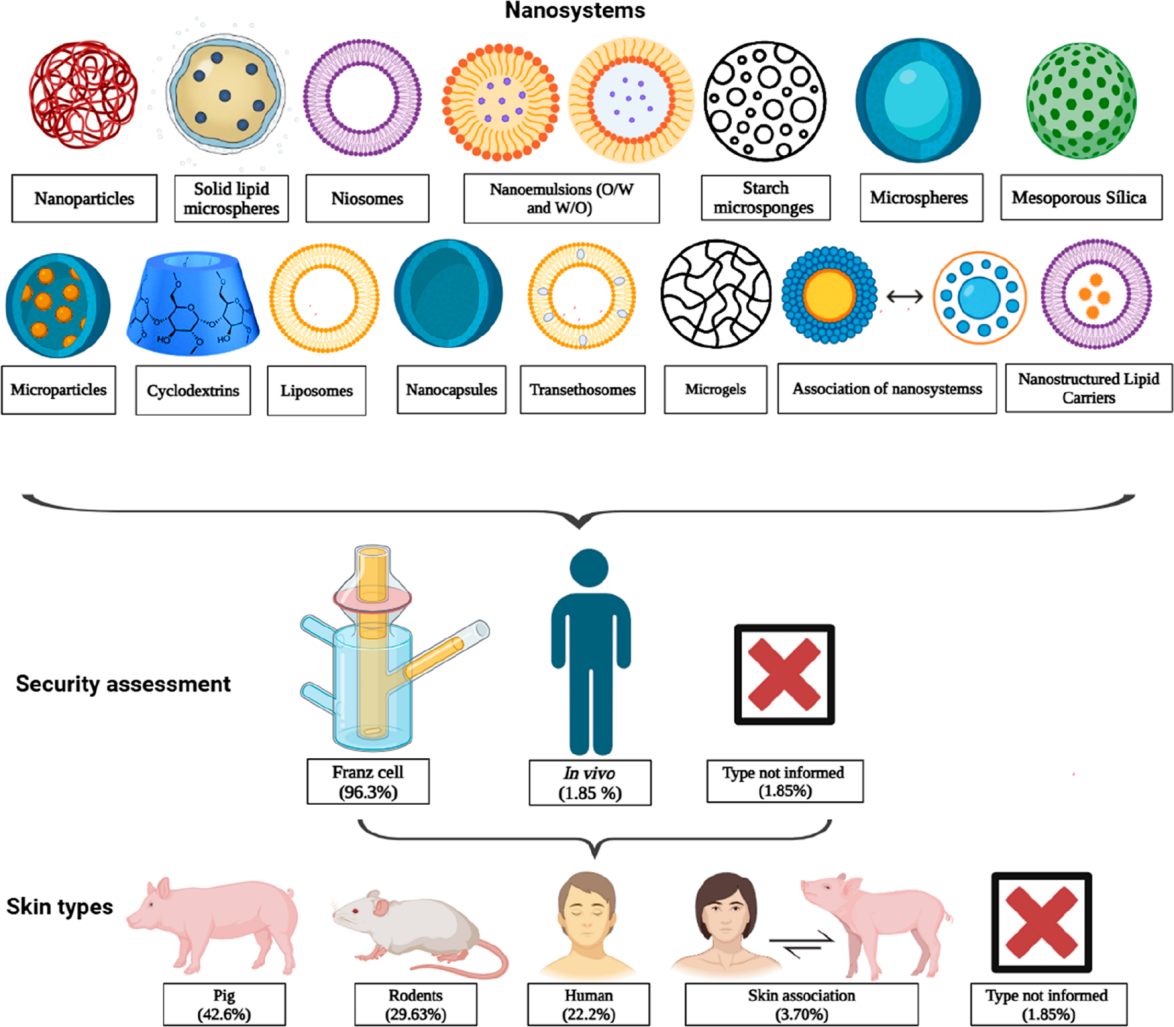
Nanosystems, safety studies, and skin types used in the trials described in this article.

Only one article used an *in vivo* methodology, with volunteers applying the product to the skin on their backs.^20^ The remaining studies were conducted in Franz cells using rodent skin (mice and rats), pig skin, and human skin, with some works combining more than one skin type. One study did not specify the evaluation model (Table 2).

**Table 2:** Assessment Methods, Results, and Conclusion of Included Studies

author/year	evaluation model	results	conclusion
Wissing, S. A.; Muller, R. H.; 2002	Franz diffusion cells with cellulose acetate membranes (*in vitro*)	OXY release is sustained and slowed when using SLN formulations instead of emulsions; the diffusion coefficient decreases with higher active loading, so more OXY remains on the skin surface where it acts as a sunscreen; SLN formulations form a film on the skin by evaporating water, trapping the sunscreen molecules within the film	SLNs can provide a sustained-release carrier system, so the sunscreen remains on the skin surface for longer where it is intended to act
Yener, G.; Incegül, T.; Yener, N.; 2003	Franz diffusion cells with cellulose acetate membrane–rat abdominal skin	in the *in vitro* study, OMC penetration rates and the amount released and penetrated decreased when OMC was in the form of microspheres; differences in penetration rates were also observed due to the vehicles; the release of free OMC was found to be higher from the oily cream and lower from the Carbopol gel in the form of lipid microspheres; in the *ex vivo* study, the penetration of free OMC was higher in oily cream, while no amount penetrated was observed in the receptor phase in the case of OMC in lipid microsphere form incorporated in an O/W emulsion	the results of this study showed that SLM can be suggested as carriers for OMC to decrease the penetration rate of this UV absorber, in addition, the choice of the appropriate vehicle plays an important role in the formulation of a sunscreen
Jiménez, M. M.; Pelletier, J.; Bobin, M. F.; Martini, M. C.; 2004	Franz diffusion cells with pig skin	after 3 and 24 h of exposure, OMC remained mainly on the skin surface; the highest concentration of OMC in the stratum corneum was obtained with the O/W and W/O emulsions; the lowest levels were found with OMC-NC emulsions; transepidermal penetration (TP) represented by the percentage of OMC in the dermis and receptor fluid was low for all formulations	the incorporation of OMC in NC decreased the release compared to the same emulsion; NCs are capable of providing a sustained-release carrier system, therefore, the sunscreen remains for longer on the skin surface where it is intended to act; from the results obtained in the study it can be deduced that the use of NC emulsions decreases the penetration of OMC in pig skin when compared to the same W/O and O/W emulsions
Luppi, B.; Cerchiara, T.; Bigucci, F.; Basile, R.; Zecchi, V.; 2004	Franz diffusion cells with porcine ear skin	systems with a low degree of substitution provided higher permeation profiles than those with a high degree of substitution, indicating the ability of these nanoparticles to allow the movement of BZP toward the skin	nanoparticles with a low degree of substitution appear to be the best candidates for improving the localization of sunscreen in the epidermis, while nanoparticles with a high degree of substitution appear to impede the percutaneous absorption of BZP
Alvarez-Roman, R.; Naik, A.; Kalia, Y. N.; Guy, R. H.; Fessi, H.; 2004	Franz diffusion cells with pig skin	the penetration of OMC into the SC of the nanoparticulate formulation was 3.4 times greater than that of an emulsion; the use of particulate drug carriers (microparticles and nanoparticles) appeared to improve drug residence in the skin without increasing transdermal transport	encapsulation of a highly lipophilic molecule (OMC) using polymeric nanoparticles significantly improved the penetration of the molecule into the stratum corneum layers, compared to a nonparticulate formulation at the same concentration, with no detectable permeation into the receptor phase
Simeoni, S.; Scalia, S.; Tursilli, R.; Benson, H.; 2006	Franz-type glass vertical diffusion cells with skin from the breast and abdomen of women aged 29–40 years	for all formulations examined, the majority of the applied sunscreen dose remained on the skin surface; appreciable levels of sunscreen permeated into the viable epidermis and dermis after application of the cyclodextrin-free OXY solution	the complexation of OXY with SBE-β-CD markedly reduces its percutaneous penetration, thus minimizing sunscreen contact with the living skin area and the potentially associated toxicological effects risks
Scalia, S.; Mezzena, M.; Iannuccelli, V.; 2007	*in vitro* by Franz-type glass diffusion cells with cellulose acetate membranes and *in vivo* tape-removal technique in women aged between 24 and 30 years	the applied dose of sunscreen that penetrated the stratum corneum was higher for nonencapsulated 4-MBC when compared to that entrapped in microparticles; the results obtained showed that the penetration of 4-MBC into the stratum corneum was lower for the emulsion containing the microparticles loaded with sunscreen compared to the formulation prepared with the free UV filter	the incorporation of 4-MBC into lipid microparticles decreases the percutaneous penetration of the sunscreen, thus minimizing its systemic absorption and the potential associated toxicological risks; an additional advantage of this effect is that more of the active sunscreen remains on the skin surface where it is intended to act
Anumansirikul, N.; Wittayasuporn, M.; Klinubol, P.; Tachaprutinun, A.; Wanichwecharungruang, S. P.; 2008	Franz vertical diffusion cells using the abdominal skin of infant mice	when encapsulated EHMC was applied to the skin, there was an even lower percentage of EHMC in the receptor medium after 24 h; since the penetration of free EHMC was significantly faster than that of encapsulated EHMC, all three particles showed controlled release of EHMC during the topically applied state	the particles could not penetrate the skin in the receptor medium throughout 24 h; in this topically applied state, all three particles showed comparable controlled release of EHMC
Wu, J.; Liu, W.; Xue, C.; Zhou, S.; Lan, F.; Bi, L.; Xu, H.; Yang, X.; Zeng, F. D.; 2009	Franz diffusion cells with porcine skin	TiO_2_ nanoparticles were detected in the stratum corneum, stratum granulosum, spinous cell layer, and basal cell layer, but not in the dermis; it is noteworthy that the ability of TiO_2_ nanoparticles to penetrate the skin depended on its size because only 4 nm TiO_2_ reached the deepest layer of the epidermis (basal cell layer)	these nanomaterials of different sizes can penetrate the skin, enter different organs, and induce various tissue damages, especially in the skin and liver of mice after chronic dermal exposure
Weiss-Angeli, V.; Bourgeois, S.; Pelletier, J.; Guterres, S. S.; Fessi, H.; Bolzinger, M. A.; 2010	Franz diffusion cells with pig flank skin (Landrace and Pietrain breeds)	filter encapsulation in NC did not modify the distribution of OMC in the skin after 3 h, since there were no significant differences between OMC-gel or OMC-NC-gel; however, after 6 h, a significant difference was observed in the dermis, the amount recovered in this compartment was higher for the control gel when compared to IMC-gel; NC accumulated mainly in the epidermis after 6 h; OMC was mainly localized in this compartment but did not cross the level of viable epidermis	these particles are promising delivery systems for the dermal administration of lipophilic molecules; NC limited the release of OMC in the deeper layers of the skin, minimizing the toxicity of the sunscreens
Senzui, M.; Tamura, T.; Miura, K.; Ikarashi, Y.; Watanabe, Y.; Fujii, M.; 2010	diffusion cell and using intact and peeled Yucatan micropig (YMP) skin as a model of injured skin	the concentration of Ti in the receptor phase was similar in all skin conditions and applied formulations; a difference was found indicating that TiO_2_ did not penetrate the skin regardless of particle size and even when the SC was removed; for depilated skin, Ti concentration in the skin after application tended to be high; in the epidermis, the concentration of TiO_2_-NPs tended to be higher than that of the control, unlike the dermis, which was not different from that of the control	TiO_2_ does not penetrate viable skin even if the particle size is less than 100 nm and the SC is damaged; however, immediately after epilation, some TiO_2_ particles penetrated relatively deeply into the empty hair follicle
Vettor, M.; Bourgeois, S.; Fessi, H.; Pelletier, J.; Perugini, P.; Pavanetto, F.; Bolzinger, M. A.; 2010	Franz diffusion cells with porcine flank skin	no OMC was recovered in the receptor compartment at any time for both simulations; when encapsulated in NP, most of the OMC was retained on the skin surface (SS) over time; in viable skin layers, percutaneous absorption of OMC using OMC-emulgel increased with exposure time; on the other hand, the amounts of OMC in the viable epidermis were almost constant for nanoparticles because of the SC barrier function that limits the diffusion of OMC from nanoparticles	the polymeric nanoparticles remained preferentially on the skin surface where OMC would act; consequently, the nanoparticles limited the penetration of OMC into the viable skin layers
Siqueira, N. M.; Contri, R. V.; Paese, K.; Beck, R. C. R.; Pohlmann, A. R.; Guterres, S. S.; 2011	Franz diffusion cells with porcine abdominal skin	the concentration of sunscreen in the stratum corneum after application of the hydrogel containing BZP loaded nanocapsules (HEC-NC-NC-B3) was almost 3 times higher than the concentration observed for the control sample; HEC-Q-NC-B3 showed a tendency to retain higher amounts of BZP in the stratum corneum compared to the HEC-NC-B3 formulation; in the viable epidermis and dermis, higher amounts of BZP were determined after the application of the formulation containing uncoated nanocapsules compared to the formulation containing free BZP	the cationic coating of nanocapsules with chitosan was efficient in maintaining the sunscreen in the superficial layers of the skin for a longer time; furthermore, the low content of BZP in the receptor fluids (Franz cells) showed the potential of this formulation to reduce the risk of systemic distribution of BZP
Marcato, P. D.; Caverzan, J.; Rossi-Bergmann, B.; Pinto, E. F.; Machado, D.; Silva, R. A.; Justo, G. Z.; Ferreira, C. V.; D uran, N.; 2011	Franz diffusion cells with human skin, plastic surgery-treated	encapsulation of BZ3 decreased its penetration into the skin; PCL nanoparticles decreased the skin permeation of BZ3 by 70% in the epidermis and dermis and by 80% in the receptor fluid; however, the skin permeation of SLN-BZ3 was not significantly different from free BZ3; this difference may be due to the flexibility of the particles	encapsulation of BZ3 in the PCL nanostructure decreased its skin permeation more than SLN-BZ3 and both nanostructures increased the sun protection factor; thus, BZ3 will remain longer on the skin surface, where it is intended to act with a higher SPF
Monteiro-Riviere, N. A.; Wiench, K.; Landsiedel, R.; Schulte, S.; Inman, A. O.; Riviere, J. E.; 2011	*in vivo*, Franz diffusion cells with porcine skin using multiple detection modalities	in the *in vitro* study, ZnO and TiO_2_ NPs did not appear to penetrate through intact healthy skin; for sunburn-damaged skin, minimal penetration of ZnO and TiO_2_ NPs into epidermal cellular elements occurred; the *in vivo* study showed that TiO_2_ penetrated deeper into the SC in both normal and UVB-exposed skin compared to ZnO	there was minimal penetration of TiO_2_ and ZnO NPs into the upper epidermal layers when applied topically in sunscreen to normal and UVB-exposed skin, with no evidence of systemic absorption
Monteiro, M. S. D. D.; Ozzetti, R. A.; Vergnanini, A. L.; de Brito-Gitirana, L.; Volpato, N. M.; de Freitas, Z. M. F.; Ricci, E.; dos Santos, E. P.; 2012	Franz diffusion cells with porcine ear skin	the free OMC formulation demonstrated a quantity of OMC in the porcine epidermis and dermis, demonstrating that OMC is capable of penetrating the skin; in the ÿ-CD/OMC formulation, a greater quantity of OMC was found in the dermis; the lipo/OMC formulation presented a greater quantity of OMC in the epidermis, which is extremely significant for antisunscreen formulations; the ÿ-CD/OMC lipo/OMS formulation showed that the quantities of OMC in the epidermis and dermis were similar, which can be attributed to the presence of both complexes in the same formulation	the lipo/OMC system demonstrated a significant increase in the quantity of OMC in the epidermis, without increasing its penetration due to the development of OMC storage
Jirova, D.; Kejlova, K.; Pikal, P.; Kasparova, L.; Safarova, K.; Kovarikova, L.; Bendová, H.; Zalabak, DE.; 2012	Franz diffusion cells with porcine skin	for particles with a mean diameter above 100 nm, more than 95% of the recovered TiO_2_ remained on the skin surface, approximately 4% of TiO_2_ was detected in the stratum corneum/epidermis and less than 1% in the dermis; for neopentyl glycol (NPG)-stabilized nanoparticles (mean particle diameter 26 nm), only 45% of the recovered TiO_2_ remained on the skin surface, while ∼45% of the TiO_2_ infiltrated the stratum corneum/epidermis	none of the tested TiO_2_ particles penetrated the receptor fluid, confirming that there is no risk of systemic exposure via the bloodstream
Hanno, I.; Anselmi, C.; Bouchemal, K.; 2012	Franz diffusion cells with pig ear skin	in all cases, epidermal penetration is considered low for NEs and NCs, while release values are much higher for NEs than for NCs	sunscreen penetrations into the skin through the epidermis for NEs and NCs were very low; this result ensures that the sunscreens will remain in the stratum corneum, the site of their activity
Gulson, B.; Wong, H.; Korsch, M.; Gomez, L.; Casey, P.; McCall, M.; McCulloch, M.; Trotter, J.; Stauber, J.; Green Oak, G.; 2012	*in vivo*, with skin from the back of volunteers; blood and urine samples were collected	small amounts of Zn from Zn oxide particles in sunscreens are absorbed by healthy human skin and are detectable in blood and urine, as observed in the outdoor test for 21 subjects	both trials using different sunscreen formulations and different UV exposures demonstrate that small amounts of Zn from ZnO particles in sunscreen are absorbed by the skin in healthy subjects and can be detected in blood and urine; however, the extra amounts of Zn added to the blood over a 5-day period are minimal compared to the body burden of Zn
Teixeira, Z.; Dreiss, C. A.; Lawrence, M. J.; Heenan, R. K.; Machado, D.; Justo, G. Z.; Guterres, S. S.; Durán, N.; 2012	Franz diffusion cells with human abdominal skin treated with plastic surgery	despite the smaller size of the NS, the NCs penetrated the SC in greater quantity, remaining in the epidermis and dermis, when compared to the NS formulation, confirming that the NC was much more deformable than the NS; BZP was not detected in the receptor solution	NCs containing a model molecule of BZP were able to distribute it in the skin and, in particular, reach the viable epidermis, without a presence in the receptor fluid
Miquel-Jeanjean, C.; Crepel, F.; Raufast, V.; Payre, B.; Datas, L.; Bessou-Touya, S.; Duplan, H.; 2012	homemade Teflon static diffusion cell with porcine membrane	in intact, irradiated, damaged, and irradiated damaged skin models, titanium was found deposited in the viable epidermis and dermis, considered bioavailable and there was no difference between samples; no titanium was detected in the receptor fluid	nanometric TiO_2_ remained in the upper layers of the SC even when the skin was previously compromised by physical or mechanical constraints and/or solar radiation
Mota, A. D. V.; de Freitas, Z. M. F.; Ricci, E.; Dellamora-Ortiz, G. M.; Santos-Oliveira, R.; Ozzetti, R. A.; Vergnanini, A. L.; Ribeiro, V. L.; Silva, R. S.; dos Santos, E. P.; 2013	vertical diffusion system with an artificial acetate membrane	the release profile for the liposome/OMC formulation was significantly different from that of the conventional free OMC formulation; the amounts of sunscreen transferred per area show that the liposome is capable of modifying the release of CAFÉ from the gel formulation; the liposome provides a lipophilic environment for OMC, which hinders diffusion into the receptor solution	liposomes could be a better carrier for OMC compared to conventional free OMC formulations, as they can modify the release of OMC and form a reservoir, thus remaining in greater quantity in the stratum corneum and minimizing systemic absorption of OMC; the liposome/OMC formulation is, therefore, a better vehicle for OMC than conventional formulations
Puglia, C.; Damiani, E.; Offerta, A.; Rizza, L.; Tirendi, G. G.; Tarico, M. S.; Curreri, S.; Bonina, F.; Perrotta, R. E.; 2014	Franz diffusion cells with skin obtained from breast reduction	the lowest fluxes of sunscreens through SCE membranes after 24 h were obtained in the case of NLC-based formulations; when incorporated into NLC, the skin permeation capacity of AVO is drastically reduced, remaining mainly at the skin surface	NLC can reduce the skin permeation of all tested sunscreens compared to NE, leading to their accumulation in the stratum corneum
Shetty, P. K.; Venuvanka, V.; Jagani, H. V.; Chethan, G. H.; Ligade, V. S.; Musmade, P. B.; Nayak, U. Y.; Reddy, M. S.; Kalthur, G.; Udupa, N.; Rao, C. M.; Mutalik, S.; 2015	*in vivo* skin permeation studies were performed in Wistar rats	NPs resulted in greater deposition of morin in the skin, indicating that the particles are also being retained to a greater extent; sunscreen creams containing nanoparticulate morin exhibited significantly (*P* < 0.05) greater retention of morin in the skin compared to creams containing pure morin	NPs produced good retention of morin in the skin
Crosera, M.; Prodi, A.; Mauro, M.; Pelin, M.; Florio, C.; Bellomo, F.; Adami, G.; Apostoli, P.; De Palma, G.; Bovenzi, M.; Campanini, M.; Filon, F. L.; 2015	Franz diffusion cells with human abdominal skin	no titanium permeation was demonstrated after 24 h of skin exposure to TiO_2_ NPs in both intact and damaged skin; in the skin, titanium was detected only in the epidermis; since the total amount of NPs was similar in both intact and damaged skin, it is assumed that lesions do not increase permeation	we found no permeation of TiO_2_ NPs in intact or damaged skin; we localized NPs in the epidermal layer, but not in the dermal layer, and the concentration in the skin was similar in both tests: skin lesions did not seem to alter the permeation of these NPs; the absence of TiO_2_ NP permeation in both intact and damaged skin suggested a low toxic potential of these nanocompounds at the skin level
Cerqueira-Coutinho, C.; Santos-Oliveira, R.; dos Santos, E.; Mansur, C. R.; 2015	skin permeation test using dorsal skin of Wistar rats	the formulation coated with the chitosan polymer (NE2) was better retained in the skin when compared to the sunscreen mixture and the nanoemulsion without chitosan (NE1)	NE2 has a greater affinity with the skin than NE1 or the sunscreen mixture; NE containing chitosan proved to be more suitable as a vehicle for substances intended to act on the skin, such as sunscreens, since sunscreens are administered topically and should not permeate the skin
Xie, G.; Lu, W.; Lu, D.; 2015	*in vivo* and *in vitro* skin permeation studies were performed on Wistar rats	TiO_2_-NPs could not penetrate SLS-damaged skin *in vitro*; the radioactive substance (125I) penetrated may be free 125I, and TiO_2_-NPs could not penetrate SLS-damaged skin *in vivo*; the results indicated that TiO_2_-NPs could not penetrate the skin *in vivo* even if the SC layer of the skin was damaged	the results indicated that TiO_2_-NPs could not penetrate through damaged skin both *in vitro* and *in vivo*; it suggested that TiO_2_-NPs should be safe when applied and in contact with the skin
Cerqueira-Coutinho, C. S.; De Campo, V. E. B.; Rossi, A. L.; Veiga, V. F.; Holandino, C.; Freitas, Z. M. F.; Ricci-Junior, E.; Mansur, C. R. E.; Santos, E. P.; Santos-Oliveira, R.; 2016	permeation assays (*ex vivo*) using Franz cells from pig ears	both NEs remained in the epidermis and dermis after 6 h; the amount of OMC retained in the epidermis by NE with chitosan was almost three times higher when compared to the amount in NE without the polymer retained in the same layer; chitosan acted on the epidermis and prevented the penetration of further OMC into the dermis	the *in vitro* permeation assay showed that NEs containing chitosan are promising carriers of OMC due to their higher retention in the epidermis, and OMCs were not found in the receptor fluid
de Oliveira, C. A.; Dario, M. F.; Sarruf, F. D.; Mariz, I. F. A.; Velasco, M. V.R.; Rosado, C.; Baby, A. R.; 2016	Franz diffusion cells with porcine ear skin	the rutin-loaded nanoparticles did not permeate the skin, since at the end of the study no flavonoids were detected in the receptor compartment; furthermore, no rutin was detected in the stratum corneum after precise and accurate quantification, or in the remaining full-thickness skin	the higher molecular weight of rutin and gelatin and the low lipophilicity explain the tendency of these nanoparticles to remain on the surface of the skin tissue
Gilbert, E.; Roussel, L.; Serre, C.; Sandouk, R.; Salmon, D.; Kirilov, P.; Haftek, M.; Falson, F.; Pirot, F.; 2016	*ex vivo* penetration and permeation study in porcine ear skin using Franz static diffusion cells	the fluxes of BP-3 when formulated in SLN and NLC suspensions did not differ significantly from those obtained with the control formulation, assuming that SLN and NLC did not increase the skin permeation of BP-3, but that free BP-3 contained in the aqueous medium penetrates the skin; when entrapped in NPLC and NC, the flux of BP-3 through the skin was significantly reduced	this study demonstrated the interest of entrapping BP-3 in polymeric lipid nanoparticles (i.e., NPLC and NC); indeed, polymeric nanoparticles were shown to significantly reduce the flux of BP-3 through porcine skin while exhibiting high SPF *in vitro*
Arslan Azizoglu, G.; Tuncay Tanriverdi, S.; Aydin Kose, F.; Ballar Kirmizibayrak, P.; Ozer O.; 2017	Franz vertical diffusion cells with abdominal skin from albino Wistar rats	elastic liposomes applied to the skin without a vehicle, such as Pickering emulsion, can penetrate and accumulate in the skin; MEL penetration was greater and faster when elastic niosomes loaded with the active were freeze-dried and combined with Pickering emulsions containing OMC	MEL can be delivered to deeper layers of the skin with elastic niosomes and accumulation of OMC in the outer layers of the skin can be achieved using Pickering emulsions in a combined formulation
Joshi, H.; Hegde, A. R.; Shetty, P. K.; Gollavilli, H.; Managuli, R. S.; Kalthur, G.; Mutalik, S.; 2018	Franz vertical diffusion cells with Wistar rat back skin	SC1, SC2 and SC3 creams (without NPs) showed noticeable permeation of naringenin into the skin; SC4 and SC5 creams (with NPs) did not show any permeation of naringenin through the skin even at the end of 12 h; this reduction in drug permeation may be due to the presence of nano ZnO and nano TiO	skin permeation profiles of different sunscreens revealed a decline in the extent of permeation and an increase in deposition of naringenin in the skin
Andreo, N.; Bim, A. V. K.; Kaneko, T. M.; Kitice, N. A.; Haridass, I. N.; Abd, E.; Lopes, P. S.; Thakur, S. S.; Parekh, H. S.; Roberts, M. S.; Grice, J. E.; Benson, H. A. E.; Leite-Silva, V. R.; 2018	Franz diffusion cells with human abdominal skin	OMC was not detected in the receptor fluid at any time point for any of the applied formulations, this compound did not permeate the entire thickness of the skin; almost all of the applied OMC was recovered from the skin surface and stratum corneum	stable SLNs containing the chemical UV filter OMC were developed
Viswanathan, K.; Vaiyamalai, R.; Bharathi Babu, D.; Mala Priyadharshini, M. L.; Raman, M.; Dhinakarraj, G.; 2018	1000 mg of cream was applied to the dermal surface of a skin sample; after 4 h, the skin sample was cut into small pieces and placed in an extraction medium	cumulative permeation and deposition indicated that a maximum of 45% penetration was recorded after 4 h; the semisolid gel made with nanoparticles increases the retention time of the drug on the skin	the semisolid gel-based formulation offers many advantages such as decreased release of the active substance into the systemic circulation and increased retention time on the skin
Cerqueira, C.; Nigro, F.; Campos, V. E. B.; Rossi, A.; Santos-Oliveira, R.; Cardoso, V.; Vermelho, A. B.; dos Santos, E. P.; Mansur, C. R. E.; 2019	Franz diffusion cells with porcine ear skin	both sunscreens were found in higher concentrations in the epidermis than in the dermis, for both formulations (F-NA and F-N2)	the increase in N2 retention in the skin is directly correlated with the positive charge of stearyl amine
Bhuptani, R. S.; Patravale, V. B.; 2019	Franz diffusion cells supplied with the dorsal portion of mouse skin	the cumulative amount of starch microsponges loaded/encapsulated with BZP (SMBNZ) that penetrated through the skin was 50% less than that of BZP cream; BZP molecules were tightly bound in the starch microsponges (SM) matrix of SMBNZ cream and very little amount penetrated through the skin	the inclusion of BNZ in SM prevented BNZ from penetrating into the deeper layers of the skin, thus reducing its systemic absorption and undesirable side effects
Daré, R. G.; Costa, A.; Nakamura, C. V.; Truiti, M. C. T.; Ximenes, V. F.; Lautenschlager, S. O. S.; Sarmento, B.; 2020	Franz diffusion cells with human skin obtained from abdominal surgery of a healthy woman	compound P0 diluted in the receptor fluid exhibited retention in the skin layers: SC, viable epidermis, and dermis, however, P0 associated with NLCs showed an increase in skin retention; compound P2 diluted in receptor fluid or associated with NLCs showed no significant difference in the amount of active retained in the skin, however, the drug retention profile in the different skin layers changed when P2 was associated with NLCs, showing 2 times less retention in SC and 1.2 times more retention in the dermis, with no significant changes in the viable epidermis	for the NLCs-P0 delivery system, the compound was retained in the upper layers of the skin; the different retention profiles of the compounds result from their different lipophilicity characteristics; the modulation in the skin retention profiles of P0 and P2 when associated with NLCs was mainly attributed to the occlusion properties of the SLN
Rodrigues, L. R.; Jose, J.; 2020	permeation apparatus modified with skin from the back of albino Wistar rats	formulation F6 showed better release due to the increased concentration of lipids and surfactants; the permeation of the active ingredient was 80.10% during the 8 h period	the release profile of the active ingredient showed improved topical retention of *Aloe vera* for a prolonged period of time
Holmes, A. M.; Kempson, I.; Turnbull, T.; Paterson, D.; Roberts, M. S.; 2020	Franz diffusion cells with human skin	there was a significant increase in zinc concentration in the skin strata after the application of ZnO NPs in artificial human sweat to intact skin; the amount of zinc permeating intact skin into the LV was relatively modest, with most of the zinc being found retained within the SC, consistent with other observations of transition metals binding to proteins within the SC; when the SC is compromised, there are significantly elevated zinc concentrations in the epidermis	ZnO NPs were present on the skin surface after 48 h, even after a thorough washout procedure, demonstrating that the dissolution process at the skin surface is not complete even when applied in the low pH formulation of artificial sweat; incomplete dissolution of ZnO NPs was also observed in biologically relevant media, this is also consistent with previous observations that ZnO NPs do not penetrate intact human skin beyond the grooves and superficial layers of the SC
Khabir, Z.; Holmes, A. M.; Lai, Y.-J.; Liang, L.; Deva, A.; Polikarpov, M. A.; Roberts, M. S.; Zvyagin, A. V.; 2021	Franz diffusion cells with human abdominal skin from female donors	high levels of zinc isotope were found within the sulci and superficial layers of the SC, which were attributed to undissolved ZnO-PEG NPs; at the same time, the skin SC contains a significant amount of keratin characterized by abundant protein sulfhydryl groups, the high binding propensity of these groups to labile zinc may contribute to the significant concentration of Zn detected in the SC	it was observed that ZnO-PEG NPs were localized and retained in the superficial layers of the SC and did not penetrate further into the LV; ZnO NPs in sunscreens are safe after topical application to intact human skin
Tomer, S.; Suh, H.; Zhou, A. G.; Yu, B.; Lewis, J.; Saltzman, M.; Girardi, M.; 2021	free AVO in dimethyl sulfoxide (DMSO) and BNP-AVO in water was applied to the surface of an *ex vivo* human skin equivalent (NativeSkin)	After 24 h, 2.3 times more free AVO penetrated the skin compared to AVO encapsulated in BNP (*P* < 0.005)	BNP encapsulation of sunscreen actives provides improved safety and performance in an optimized sunscreen formulation
Daneluti, A. L. M.; Guerra, L. O.; Velasco, M. V. R.; do Rosário Matos, J.; Baby, A. R.; Kalia, Y. N.; 2021	Franz diffusion cells with swine ear skin and human skin	in this experiment, the commercially available stick containing nonincorporated UV filters and the stick with incorporated filters were used; SBA-15 was able to reduce skin penetration (AVO and OXY) and transdermal permeation of UV filters (OXY) in swine and human skin. The incorporated filters applied to human and swine skin significantly reduced OXY permeation and skin deposition of OXY and AVO compared to the commercially available stick product	the biodistribution results demonstrated that encapsulation with SBA-15 decreased the amounts of AVO and OXY present in the deep layers of human and swine skin (dermis)
Basto, R.; Andrade, R.; Nunes, C.; Lima, S. A. C.; Reis, S.; 2021	Franz diffusion cells with pig ear skin	higher retention of NIA in the skin layers can be verified in the case of nanogels containing jojoba oil TE, which showed a more controlled permeation between the nanogels; comparing with the data of free NIA, it is clear that all formulations have a more controlled permeation capacity since the rate of NIA appearance is less than half in all cases	among all formulations, it is clear that TEs are more effective in retaining NIA in the skin layers for the intended topical effect
Kaur, J.; Anwer, M. K.; Sartaj, A.; Panda, B. P.; Ali, A.; Zafar, A.; Kumar, V.; Gilani, SJ.; Kala, C.; Taleuzzaman, M.; 2022	Franz diffusion cells using *ex vivo* rat skin	the percentages of cumulative active permeated through rat skin were higher for ZnO-MJE-NPs-opt	the semisolid gel made with nanoparticles increases the retention time of the drug on the skin; the gel-based semisolid offers many advantages such as decreased activity in systemic circulation and longer retention time on the skin
Ghazwani, M.; Hani, U.; Alqarni, M.H.; Alam, A.; 2023	Wistar rat abdominal skin in Franz diffusion cells	the MA-AgNP gel significantly increased MA transport in the epidermal layers compared to the conventional MA-loaded gel (MA-CFG); furthermore, the dermis exhibited a significantly higher area under the curve (AUC) of MA than the MA-CFG gel; the results showed that the MA-AgNP gel increased the retention of the active ingredient in both layers	the MA-AgNP gel penetrated deeper into the dermis and epidermis than the MA-CFG gel, demonstrating that the developed gel formulation can be useful for topical applications where greater penetration and retention of the active ingredient are sought
Ma, Q.; Zhang, Y.; Huangfu, Y.; Gao, S.; Zhou, C.; Rong, H.; Deng, L.; Dong, A.; Zhang, J.; 2023	Franz diffusion cells in swine skin	compared with free DHHB, MSN-DHHB was able to slow but not prevent DHHB skin penetration; virtually no DHHB skin penetration from MSN-DHHB@SiO_2_ was measured over 24 h, indicating that the silica layer completely prevented DHHB penetration into the skin	MSN-DHHB@SiO_2_ provided a promising method for solving the skin penetration problem of organic filters
Sousa, I. P.; Landim, A. C. T.; Ribeiro, B. C. C.; Cintra, E. R.; Silva, L. M.; Nascimento, T. L.; Lima, E. M.; Silva, L. A. D.; Diniz, D. G. A.; 2023	Franz diffusion cells in swine ear skin	encapsulation in NLCs promoted greater cutaneous retention of AVO, presenting significantly higher retention at the skin surface compared to the concentration that penetrated the stratum corneum; semisolid formulations containing nanocapsules provided greater retention in the stratum corneum compared to semisolids containing free BZP	NLCs showed greater retention on the skin surface when compared to AVO that penetrated the stratum corneum
de Araújo, M. M.; Schneid, A. C.; Oliveira, M. S.; Mussi, S. V.; de Freitas, M. N.; Carvalho, F. C.; Bernes Junior, E. A.; Faro, R.; Azevedo, H.; 2024	*ex vivo* in human skin obtained in optional abdominoplasty surgeries using the DHC-6T dry heat transdermal system (Franz cells)	equivalent permeation of all three filters was observed in the epidermis and dermis for the SC-NLC and emulsion samples, and no filters were detected in the receptor fluid	therefore, the nanometric dimensions of SC-NLC did not lead to increased skin absorption of the filters, making it safe for subsequent topical use; the observed permeation for SC-NLC was possibly hampered by repulsion between the negative charges on the carrier surface (as indicated by the ζ potential data) and the lipid composition of the stratum corneum
Wang, W.; He, Q.-T.; Chen, Y.-F.; Wang, B.-H.; Xu, W.-Y.; Liu, Q.-L.; Liu, H.-M.; 2024	Franz diffusion cells in swine dorsal skin	the EHMC-pEDGMA microgel demonstrated the ability to reduce the penetration of EHMC into human skin; the decreased penetration of EHMC-pEGDMA was attributed to the macromolecular nature of the pEDGMA polymer, which was characterized by large particle sizes	this microgel met the necessary criteria, which included minimal penetration
Zhang, J.; Zhang, S.; Yan, C.; Bi, J.; Han, X.; Liu, H.; 2024	Franz diffusion cells in swine creases	the SiO_2_-PDA nanoparticles exhibited stable adhesion to the skin surface with little skin penetration; the limited penetration of these particles can be attributed to their high adhesion properties and electrostatic repulsion with the skin	SiO_2_-PDCA nanoparticles are a potential candidate for tinted sunscreens or other biomedical applications
Tarantini, A.; Jamet-Anselme, E.; Lam, S.; Haute, V.; Suhard, D.; Valle, N.; Chamel-Mossuz, V.; Bouvier-Capely, C.; Phan, G.; 2024	swine ears in Franz diffusion cells	Ti accumulated in the receptor fluid as a function of time in excoriated skin exposed to Ti ions, but not in the receptor fluid of healthy skin; regarding TiO_2_ NPs, Ti levels were below the detection limit at all sampling times, meaning that the applied dose of TiO_2_ was able to permeate both skin types under our experimental conditions; no greater permeation of TiO_2_ NPs through excoriated skin compared to healthy skin was observed	TiO_2_ NPs can diffuse through damaged and healthy skin in small amounts after exposure; the majority of applied TiO_2_ NPs (60%) remained on the surface of healthy skin after 24 h; despite the loss of the most superficial layers of the epidermis, the NPs can still be retained in the deeper layers of the epidermis or dermis
Shoaib Khan, H. M.; Butt, H.; Sohail, M.; Rehman, S.; Ramzan, N.; Abbas Malik, H. M.; 2025	Franz diffusion cells using *ex vivo* mouse abdominal skin	no traces of rutin were found in the pure rutin gel; the R concentration found in the gel’s receptor medium was likely due to the change in rutin’s crystalline structure to an amorphous form after conjugation with silica nanoparticles; the zinc oxide gel without nanoparticles also showed no absorbance in aliquots taken from the receptor chamber	the rutin-silica nanocomposite has negatively charged silanol groups, therefore, the negatively charged skin surface becomes a barrier in the transepidermal delivery of rutin, confining the rutin NPs in the topical layers of the skin
Gui, H.; Liu, H.; Cai, Y.; Nian, J.; Liu, L.; Song, Y.; Kye, S.; Zuo, S.; Yao, C.; 2025	Franz diffusion cells in fresh Balb/c mouse dorsal skin	Al(OH)_3_@TiO_2_ NPs exhibited significant permeation through the epidermis and accumulation in the subcutaneous layers; in stark contrast, minimal penetration of TiO_2_HA NPs was observed, with most particles retained on the epidermal surface	biosafety was demonstrated with *in vitro* penetration profiles on the skin of BALB/c mice, confirming the retention of most nanoparticles on the epidermal surface of TiO_2_ HA cream
Li, Z.; Chen, L.; Qiu, X.; 2025	Franz diffusion cells in swine skin	microcapsules, due to their micrometer-sized particles, demonstrated negligible skin penetration; for the five microcapsule samples with different loading capacities and particle sizes, the cumulative permeability remained nearly zero at 12 h	microcapsules could maintain long-term stability, thus mitigating the potential health risks associated with chemical sunscreens; thus, encapsulating AVO in lignin microspheres offers a promising approach for sunscreen formulations, as they ensure minimal skin penetration, enhancing their safety profile and making them safer and more effective

Some studies (*n* = 52) concluded that nanosystems are capable of providing a sustained-release carrier system, in which the sunscreen remains longer in the superficial layers of the skin, minimizing systemic absorption and sunscreen toxicity (Table 2).

One article reported that small amounts of Zn from Zn oxide particles in sunscreens could be detected in blood and urine. However, the extra amounts of Zn added to the blood over a 5 h period are minimal compared to the body’s Zn burden. Similarly, research has shown that elastic niosomes can penetrate deeper layers of the skin.

Another study reported that titanium dioxide (TiO_2_) nanoparticles (NPs) can diffuse through both damaged and healthy skin in small quantities. However, the majority of the applied nanoparticles (60%) remained on the surface of healthy skin after 24 h. Even after the loss of the most superficial layers of the epidermis, it was observed that some of the NPs could be retained in deeper layers such as the epidermis and dermis.

## Discussion

Excessive sun exposure represents a global public health problem, being directly associated with the development of skin cancer.^24^ Therefore, sunscreens have become essential products for photoprotection.^25^ In this context, nanotechnology has emerged as a promising strategy, offering innovative approaches for the encapsulation of UV filters in different nanosystems. These systems have the potential to enhance photoprotection, improve the retention of active ingredients on the skin surface, and consequently reduce systemic permeation, minimizing toxicological risks.^14,26^ Nevertheless, concerns regarding the safety of nanocarriers remain, particularly related to their skin behavior, biodistribution, and potential long-term adverse effects.^26^ This review sought to map studies evaluating the safety of sunscreens delivered in nanosystems, each with its own unique characteristics and objectives.

The articles included in this review indicate that nanocarriers can improve both the safety and the efficacy of sunscreens. These parameters are closely related to the photoprotective performance and exposure profile of the active ingredients. Nanocarriers promote a more uniform distribution on the skin surface, ensuring continuous and homogeneous coverage against UVA and UVB radiation, which is directly associated with an enhanced efficacy. Additionally, they contribute to improved photostability by preserving the ability of UV filters to absorb or reflect radiation throughout the period of sun exposure. Another key advantage is the controlled release and enhanced skin retention of active compounds, which limits penetration into deeper skin layers and reduces potential systemic absorption, directly impacting formulation safety.^1,4,16,17,19,20,29,33,41,46,51^

Consequently, nanocarriers increase the delivery of active ingredients to the desired site of action while maintaining biodegradability and biocompatibility along with high encapsulation efficiency. These features collectively contribute to improved performance and reduced toxicological risks when compared to conventional sunscreen formulations.^1,11,19,20,29,33,35,41,46,48,55,56^

Their safety is generally assessed based on skin permeation studies. Among the methodologies employed, the use of Franz diffusion cells, recommended by the FDA, stands out as a widely used *in vitro* tool.^1,3,4,11,13,16,17,19–22,27–63^ Diffusion cells are the most widely used *in vitro* screening tools for evaluating the permeation of drugs or nanoparticles through the skin. Introduced by Dr. Thomas J. Franz in 1970, these cells are generally composed of a donor chamber, a receptor chamber, and a semipermeable membrane that separates both chambers, allowing drug diffusion.^62^

During the development stages of studying membrane kinetics, quantification of skin permeation is a crucial step.^63^ However, there are numerous variables in this methodology, such as agitation, temperature, and dosage, which consequently result in a high degree of variability between replicated experiments, but, even so, it is a very widespread method during the formulation development stages to study kinetics through membranes.^64^

In the *in vitro* studies included in this review, porcine skin was frequently selected as the permeation barrier due to its structural and compositional similarities to human skin.^4,17,20,21,27,28,30,31–36,38,40,44–46,51,53,56–58,61^ Pig ear skin has a comparable thickness of the stratum corneum and epidermis, as well as similar follicular density, making it a well-established surrogate model in skin permeation studies.^65^

Rodent skins are also used, for example, rats and mice^1,3,5,11,17,29,32,43,48,49,52,54,59,60,66,67^ employed due to their availability, ease of handling, small size, and lower cost.^2^ However, comparative studies have demonstrated that rodent skin is significantly more permeable than human skin (sometimes by up to 10-fold), which may lead to an overestimation of skin penetration and limit its translational relevance.^65,68^

Although animal-derived membranes are widely used, the application of human skin models is strongly encouraged due to their superior physiological relevance^22,35,39,41,42,48,50,53,57,69^ presenting specific structural and biochemical characteristics, such as stratum corneum thickness, lipid composition, follicular density, and organization of cell junctions. These factors are crucial for evaluating the efficacy and safety of photoprotectors.^65^

*In vitro* studies, such as cytotoxicity and skin permeation assays using Franz diffusion cells, synthetic or nonsynthetic skin models, are widely used due to their reproducibility, lower cost, and ethical advantages; however, these approaches have limitations when compared to real physiological conditions.^1,19,20,28,31,33,36,41,42,44^

Nevertheless, *in vivo* models allow for a more integrated assessment not only of photoprotective efficacy but also of safety, including potential inflammatory effects and systemic absorption, but are less frequent due to ethical restrictions, high costs, and greater experimental complexity.^1,2,23,51,71,72^ The combination of these *in vitro* and *in vivo* models helps to understand the biological behavior of different nanosystems, reducing methodological biases and strengthening the interpretation of results, which improve the scientific quality and translational relevance of the studies analyzed.

Formulations with inadequate photoprotective efficacy may result in insufficient protection against UVA and UVB radiation, increasing the risk of erythema, sunburn, premature photoaging, cumulative DNA damage, and, ultimately, skin cancer development.^3^ From a safety perspective, sunscreens that fail to adequately control skin penetration may promote systemic absorption of UV filters, raising concerns related to endocrine disruption, immunological reactions, and other long-term toxicological effects.^10^ These risks underscore the importance of developing sunscreen formulations that are both effective and safe.

These limitations become even more relevant in the context of nanostructured sunscreens, which are designed to remain predominantly in the superficial layers of the skin, minimizing systemic exposure. In this scenario, the adoption of New Approach Methodologies (NAMs), including three-dimensional skin models, assays based on human cells, computational tools, and omics approaches, represents a significant advance.^71^

Such methodologies are strongly encouraged by international regulatory agencies such as the European Medicines Agency (EMA), the European Chemicals Agency (ECHA), and the Brazilian Health Regulatory Agency (ANVISA), and are aligned with ethical principles and animal welfare requirements, especially in regions where the use of animals in cosmetic testing is restricted or prohibited.^18,70,72^ Nevertheless, standardizing experimental models for the toxicological evaluation of nanostructured sunscreens remains a substantial challenge, mainly due to the limitations of currently available methods for quantifying and tracking nanosystems in biological tissues, as well as the high costs and ethical requirements associated with advanced toxicological assays.^2,32,44^ Despite these limitations, such approaches are indispensable for guiding future research, supporting regulatory advancement, and ensuring the safe development of nanotechnology-based photoprotective formulations.

The vast majority of studies included in this review^1,3–5,11–13,17–20,22,27–61,67,69^ concluded that nanosystems limited the permeation of sunscreens into the deeper layers of the skin, preferentially remaining on the skin surface, where the sunscreens should act, minimizing their toxicity.

Furthermore, the incorporation of nanocarriers into sunscreen formulations offers clear advantages over conventional systems. An example is the study by Daneluti et al.,^31^ which investigated whether the UV filters OXY, AVO, and OMC, incorporated into SBA-15 mesoporous silica, met safety requirements by significantly reducing skin permeation and deposition. The authors demonstrated that OXY-, AVO-, and OMC-loaded SBA-15 mesoporous silica stick formulations significantly reduced OXY permeation of the OXY and AVO skin compared to that of a commercially available stick product. In conventional formulations, these filters (AVO, OXY, and OMC) present limitations such as photochemical instability, potential systemic skin penetration, and an increased risk of irritation or long-term adverse effects.^18,73,74^ Therefore, encapsulation in nanocarriers emerges as an effective strategy to overcome these limitations, contributing to improved safety, efficacy, and overall performance of photoprotective formulations compared with systems without nanotechnology.

Ghazwani and colleagues,^54^ when developing a sunscreen gel using silver nanoparticles loaded with methyl anthranilate (MA) (MA-AgNPs), observed that MA transport in the epidermal layers increased compared to the conventional gel formulation loaded with MA, demonstrating that the MA-AgNPs gel was able to increase retention in the dermal layers.

Furthermore, studies evaluating combinations of nanosystems containing UV filters also demonstrated benefits, such as minimal penetration^33^ and a significant increase in the amount of filter in the epidermis without increasing its penetration.^2,40,66^ One article reported that small amounts of Zn from ZnO particles in sunscreens were detectable in blood and urine.^23^ However, ZnO nanoparticles are commonly used for dermal applications due to their easy visualization through a microscope on the skin, presenting primarily in the stratum corneum without any toxic effects.^18^

Similarly, Tarantini et al.^57^ investigated TiO_2_ nanoparticles and observed that they could also penetrate both intact and damaged skin, albeit in small quantities. Although the majority of the applied nanoparticles (approximately 60%) remained retained on the surface of healthy skin after 24 h. Even with the removal of the most superficial layers of the epidermis, it has been found that NPs can still accumulate in the deeper layers of the epidermis or even in the dermis.

Research has shown that elastic niosomes can penetrate the deeper layers of the skin,^16^ but it is generally known that the performance of a sunscreen formulation depends not only on the physicochemical properties of the filters but also on the carrier used to trap and distribute them.^73^

The main gap identified in this review relates to the safety of these nanosystems’ skin retention. This characteristic is essential to ensure the desired localized effect, especially for sunscreens, which must act only on the superficial layers: epidermis and dermis.^14,26^ Although many studies have suggested retention or low permeation, few simultaneously address the toxicity, efficacy, and stability aspects of the formulations, highlighting the need for more robust and integrated investigations.

The behavior of different nanosystems is associated with their physicochemical properties, including controlled particle size, lipid or polymeric matrix, and favorable interactions with the stratum corneum.^1,4^

From a toxicological point of view, comparative studies suggest that SLNs and NLCs generally have a better biocompatibility profile, since they use physiologically compatible lipids, which reduces the risk of cytotoxicity and inflammatory responses.^1,13,41^ In contrast, polymeric nanocapsules and inorganic systems, such as TiO_2_, ZnO, and mesoporous silica nanoparticles, may exhibit greater variability in toxicity profiles, depending on factors such as material composition, surface charge, and degree of degradation.^27,30–32,46,51^

In this context, a comparative analysis of the toxicity profiles between different nanosystems shows that nanotechnology should not be evaluated as a homogeneous concept but rather as distinct biological behaviors. Understanding these differences is fundamental for the development of safer and more effective photoprotective formulations as well as for guiding regulatory decisions and future research focused on long-term safety.

Conducting robust toxicity studies is paramount to strengthening the safe use of nanostructured sunscreens, given that different nanosystems may exhibit distinct behavior in terms of cytotoxicity, skin penetration, and potential systemic absorption.^2,4,30,42,46^ However, the available literature is still relatively scarce, especially regarding *in vivo* and long-term clinical studies, and this may be related to methodological and regulatory challenges, since the regulation of nanotechnology-based sunscreens differs among the main jurisdictions (Food and Drug Administration (FDA), European Union (EU), Australia, Cosmetics Directive of the Association of Southeast Asian Nations (ASEAN), and others).^2,4,74^

Although the present study shows advances in the application of nanotechnology to sunscreens, there is significant diversity in the methodologies used to evaluate different nanosystems.

This review presents some limitations that should be considered in the interpretation of the results, such as the methodological heterogeneity of the included studies, especially regarding the experimental models employed (*in vitro* and *ex vivo*), which do not fully reproduce the physiological conditions of human skin *in vivo*. In addition, the wide variability in the types of nanocarriers makes direct comparisons between studies difficult and may significantly influence the outcomes related to skin permeation, dermal retention, and safety of sunscreens, suggesting a possible publication bias. The scarcity of clinical studies and long-term evaluations limits more robust conclusions about the chronic safety and translational relevance of nanosystems applied to photoprotective formulations.

Therefore, the interpretation of the data must carefully consider the experimental model adopted, reinforcing the need for more standardized and complementary approaches to evaluate the efficacy and safety of nanostructured sunscreens.

Although the included studies employed systems that can be quite different (nanoparticles, nanosystems, nanoemulsions, microspheres, liposomes, among others) and varied biological models (humans, pigs, rodents), the conclusions of this review suggest that these systems represent significant advances over conventional formulations, especially due to their potential to increase the safety of sunscreens.

## Conclusions

The results of this exploratory review suggest that incorporating sunscreens into nanostructured systems can improve photostability and modulate skin penetration, compared to conventional formulations. Sunscreen formulations with inadequate performance may offer insufficient protection against UVA and UVB radiation, increasing the risk of erythema, sunburn, photoaging, cumulative DNA damage, and skin cancer. Furthermore, the lack of control over skin penetration may favor the systemic absorption of UV filters, raising concerns about potential long-term endocrine, immunological, and toxicological effects. Therefore, the development of sunscreens that combine high efficacy with safety becomes essential.

The 54 studies included in this review consistently corroborate the conclusions presented, as they cover a wide variety of nanosystems, UV filters, experimental models, and safety and efficacy assessment strategies. However, more studies are needed, especially safety studies, as they require a longer residence time in the dermal layers, preventing systemic absorption. The use of nanotechnology has been observed in scientific articles due to the improvements it offers not only to formulations but also to UV filters. Therefore, the combination of nanostructures and UV filters offers excellent opportunities for the cosmetics industry.

## Methods

### Study Design

This scoping review was conducted following Joanna Briggs’ guidelines for mapping, describing, and categorizing available information on the safety of formulations using nanotechnology for photoprotection.

The selection steps for the studies included in this review were conducted based on the Preferred Reporting Items for Systematic Reviews and Meta-Analyses (PRISMA) statement and its extension for Scoping Reviews (PRISMA-ScR).^15^ This research protocol is registered in the Open Science Framework database, available at https://osf.io/xbpyg/?view_only=4cfbe5bd4fde400bb41f54b80d22ac8b (2025-06-10).

### Stakeholder Involvement

This review involved the participation of two professionals (AFONSO, MS; SOUZA, PM; SANTOS, AL dos) with experience in developing scoping reviews, as well as two professionals specializing in formulations (FREITAS, ZMF; DO CARMO, FA).

### Research Question

This scoping review was guided by the following question: “How have nanosystems been applied in sunscreens and what is the evidence regarding their safety?” The research question follows the acronym Population, Concept, and Context (PCC):

- Population: nanosystems applied to topical formulations.
- Concept: evaluation of improved safety in photoprotective formulations through nanotechnology.
- Context: photoprotective formulations used in cosmetic products.

### Search Strategy for Identifying Studies

The complete strategy for each database is described in Table 3 and was developed using the following Medical Subject Headings (MeSH), Health Descriptors (DeCS), alternative terms, and keywords:

**Table 3:** Search Strategy for the Articles

database	keywords
MEDLINE (Pubmed), Embase, Lilacs (BVS), Scopus, and Web of Science	(Nanoparticle Drug Delivery System OR nanosystems OR “nanosystems” OR “Nano-Drug” OR “Nano Delivery System” OR nanoemulsion OR niosomes OR liposomes OR nanoparticles) AND (“sunscreening agents” OR sunscreens) AND (“*in vitro*“ OR “*ex vivo*“)
Google Scholar	Nanoparticle Drug Delivery System; Nanosystems; Nano Systems; Nano-Drug; Nano Delivery System; Nanoemulsion; Niosomes; Liposomes; Nanoparticles; Sunscreening Agents; Sunscreens; *In Vitro*; *Ex Vivo*

MeSH: Liposome; Transferosomes; Niosomes; Niosome; Nanoparticle; Nanocrystalline Materials; Nanocrystalline Material, Nanocrystals, Nanocrystal, Sunscreen; Agents, Sunscreening; Sunscreens.

DeCS: Liposomes; Nanoparticles; Sunscreening Agents.

Alternative terms for DeCS: Liposomes; Niosomes; Nanocrystalline Materials; Nanocrystals; Sunscreen Agents; Sunscreen; Sunscreens.

Keywords: Nanoemulsions, Niosomes, Liposomes, Nanoparticles, Sunscreens, In vitro and in vivo.

### Electronic Databases for Study Identification

The search was conducted up to June 10, 2025, in five electronic databases: MEDLINE (PubMed), Embase, BVS (Biblioteca Nacional em Sade Brasil), Scopus (Elsevier), and Web of Science (WoS, Clarivate Analytics), without restrictions on year and language.

#### Other Search Resources for Study Identification

The search strategy was also adapted to gray literature including Google Scholar. Manual searches of the included studies were also performed to find as much material as possible for this review.

### Eligibility Criteria

Primary and secondary studies, conducted *in vivo* and *in vitro*, that addressed the influence of nanosystems on the safety of sunscreens, especially regarding permeation and skin retention, were included. No restrictions were applied regarding the year of publication, country of origin, or location of the studies. Studies that did not simultaneously address nanosystems, sunscreens, and safety-related aspects were excluded. Literature reviews, book chapters, conference abstracts, and letters to the editor were also excluded.

### Eligibility Determination

References were managed and selected using the Rayyan web application (Rayyan, Intelligent Systematic Review, Rayyan), where duplicates were automatically removed. Reviewers underwent a calibration process before determining eligibility, achieving a Cohen’s Kappa coefficient of 0.97.

Titles and abstracts were independently assessed by three reviewers (AFONSO, MS; SOUZA, PM; and SANTOS, AL) to verify their eligibility. Subsequently, the same reviewers independently read the entire article to confirm eligibility within the guidelines described above. Discrepancies were resolved by consensus from a third reviewer when necessary.

### Data Extraction

Data from the included studies were independently extracted by three reviewers (AFONSO, MS; SOUZA, PM; SANTOS, AL). The information was organized in a Microsoft Excel spreadsheet. The same reviewers independently performed data extraction. Discrepancies were resolved through discussion and consensus. The reviewers were calibrated by extracting at least three documents of different quality levels and reaching consensus. This procedure was repeated until the reviewers were able to extract the data correctly and in a standardized manner. For this scoping review, the following data were extracted: Study characteristics: country, study design, bibliometric information (Digital Object Identifier (DOI), authors, year of publication, title, and country of the article).

- Study characteristics: country, study design, bibliometric information (DOI, authors, year of publication, title, and country of the article);
- Intervention characteristics: evaluation method and nanosystem used;
- Outcome characteristics: performance of the formulation using nanosystems.

## Abbreviations

4-MBC: 4-methyl benzylidene camphor
Al­(OH)_3_: aluminum hydroxide
ANVISA: Brazilian Health Regulatory Agency
ASEAN: Association of Southeast Asian Nations
AVO: avobenzone
BNP: bioadhesive nanoparticles
BZP: benzophenone-3
CD: cyclodextrins
DHHB: diethylamino hydroxybenzoyl hexyl benzoate
DMSO: dimethyl sulfoxide
EHMC: 2-ethylhexyl-4-methoxycinnamate
EHT: ethylhexyl triazine
EMA: European Medicines Agency
EU: European Union
ECHA: European Chemicals Agency
FAs: fatty acids
FDA: Food and Drug Administration
GNPs: gelatin nanoparticles
HA: hyaluronic acid
HP-c-CD: hydroxypropyl-cyclodextrin
LMs: lipid microparticles
MA: methyl anthranilate
MEL: melatonin-loaded elastic niosomes
MSN: mesoporous silica nanoparticles
MSNTiO_2_: TiO_2_ nanocrystals
NAMs: new approach methodologies
NC: nanocapsule
NIA: niacinamide
NLCs: nanostructured lipid carriers
NPG: neopentyl glycol
NPLC: polymeric lipid carriers
NPs: nanoparticles
NS: nanospheres
O/W: oil/water
OXY: oxybenzone
PCL: poly­(caprolactone)
PLA: poly­(d,l-lactide)
pEDGMA: poly­(ethylene glycol dimethacrylate)
PVA: poly­(vinyl alcohol)
RP: retinyl palmitate
SBE-β-CD: sulfobutyl ether-β-cyclodextrin
SiO_2_-PDA: polydopamine silica nanoparticles
SLMs: solids lipid microspheres
SLN: solid lipid nanoparticle
SM: starch microsponges
SPF: sun protection factor
SS: skin surface
TEs: transethosomes
TEOS: tetraethyl orthosilicate
TiO_2_: titanium dioxide
TiO_2_-NPs: titanium dioxide nanoparticles
TP: transepidermal penetration
UV: ultraviolet
UVB: ultraviolet B
W/O: water/oil
YMP: Yucatan micropig
ZnO: zinc oxide
ZnO-MJE: zinc oxide-Manjistha nanoparticles extract

## References

[1] Wissing S. A., Müller R. H. (2002). Solid lipid nanoparticles as carrier for sunscreens:
in vitro release and in vivo skin penetration. J. Controlled Release.

[2] Monteiro-Riviere N. A., Wiench K., Landsiedel R., Schulte S., Inman A. O., Riviere J. E. (2011). Safety Evaluation
of Sunscreen Formulations Containing
Titanium Dioxide and Zinc Oxide Nanoparticles in UVB Sunburned Skin:
An In Vitro and In Vivo Study. Toxicol. Sci..

[3] Ricci-Junior E., Dellamora Ortiz G. M., Pereira dos Santos E., de Carvalho Varjao Mota A., Antonio Ozzetti R., Luiz Vergnanini A., Santos-Oliveira R., Santos Silva R., Lira Ribeiro V., Maria Faria de Freitas Z. (2013). In vivo and
in vitro evaluation of octyl methoxycinnamate liposomes. Int. J. Nanomed..

[4] Cerqueira C., Nigro F., Campos V. E. B., Rossi A., Santos-Oliveira R., Cardoso V., Vermelho A. B., dos Santos E. P., Mansur C. R. E. (2019). Nanovesicle-based formulations for
photoprotection:
a safety and efficacy approach. Nanotechnology.

[5] Cerqueira-Coutinho C., Santos-Oliveira R., dos Santos E., Mansur C. R. (2015). Development of a
photoprotective and antioxidant nanoemulsion containing chitosan as
an agent for improving skin retention. Eng.
Life Sci..

[6] Micha J. P., Bohart R. D., Goldstein B. H. (2025). A review of sunscreen in the prevention
of skin cancer. J. Oncol. Pharm. Pract..

[7] Wang J., Pan L., Wu S., Lu L., Xu Y., Zhu Y., Guo M., Zhuang S. (2016). Recent Advances
on Endocrine Disrupting Effects of
UV Filters. Int. J. Environ. Res. Public Health.

[8] Iannacone M. R., Hughes M. C. B., Green A. C. (2014). Effects of sunscreen on skin cancer
and photoaging. Photodermatol., Photoimmunol.
Photomed..

[9] Moammeri A., Chegeni M. M., Sahrayi H., Ghafelehbashi R., Memarzadeh F., Mansouri A., Akbarzadeh I., Hejabi F., Abtahi M. S., Ren Q. (2023). Current advances in
niosomes applications for drug delivery and cancer treatment. Mater. Today Bio.

[10] Nascimento
Júnior J. A. C., Santos A. M., Oliveira A. M. S., Santos A. B., de Souza Araújo A. A., Aragón D. M., Frank L. A., Serafini M. R. (2024). The Tiny Big Difference: Nanotechnology
in Photoprotective Innovations – A Systematic Review. AAPS PharmSciTech.

[11] Yener G., Incegül T., Yener N. (2003). Importance of using
solid lipid microspheres
as carriers for UV filters on the example octyl methoxy cinnamate. Int. J. Pharm..

[12] Bhuptani R. S., Patravale V. B. (2019). Starch
microsponges for enhanced retention and efficacy
of topical sunscreen. Mater. Sci. Eng., C.

[13] Daré R. G., Costa A., Nakamura C. V., Truiti M. C. T., Ximenes V. F., Lautenschlager S. O. S., Sarmento B. (2020). Evaluation of lipid nanoparticles
for topical delivery of protocatechuic acid and ethyl protocatechuate
as a new photoprotection strategy. Int. J. Pharm..

[14] Nascimento
Junior J. A. C., Santos A. M., Oliveira A. M. S., Santos A. B., Araujo A. A. d. S., Frank L. A., Serafini M. R. (2024). Use of Nanotechnology
Applied to Sunscreens: Technological Prospection Based on Patents. J. Drug Delivery Sci. Technol..

[15] McGowan J., Straus S., Moher D., Langlois E. V., O’Brien K. K., Horsley T., Aldcroft A., Zarin W., Garitty C. M., Hempel S., Lillie E., Tunçalp Ö., Tricco A. C. (2020). Reporting scoping reviews – PRISMA ScR extension. J. Clin. Epidemiol..

[16] Arslan
Azizoglu G., Tuncay Tanriverdi S., Aydin Kose F., Ballar Kirmizibayrak P., Ozer O. (2017). Dual-Prevention for UV-Induced Skin
Damage: Incorporation of Melatonin-Loaded Elastic Niosomes into Octyl
Methoxycinnamate Pickering Emulsions. AAPS PharmSciTech.

[17] Basto R., Andrade R., Nunes C., Lima S. A. C., Reis S. (2021). Topical Delivery
of Niacinamide to Skin Using Hybrid Nanogels Enhances Photoprotection
Effect. Pharmaceutics.

[18] Viswanathan K., Vaiyamalai R., Bharathi babu D., Mala Priyadharshini M.
L., Raman M., Dhinakarraj G. (2018). Ketoconazole-conjugated ZnO nanoparticles
based semi-solid formulation and study their impacts on skin disease. IET Nanobiotechnol..

[19] Scalia S., Mezzena M., Iannuccelli V. (2007). Influence
of solid lipid microparticle
carriers on skin penetration of the sunscreen agent, 4-methylbenzylidene
camphor. J. Pharm. Pharmacol..

[20] Jiménez M., Pelletier J., Bobin M. F., Martini M. C. (2004). Influence of encapsulation
on the in vitro percutaneous absorption of octyl methoxycinnamate. Int. J. Pharm..

[21] Wang W., He Q.-T., Chen Y.-F., Wang B.-H., Xu W.-Y., Liu Q.-L., Liu H.-M. (2024). Anti-UV
Microgel Based on Interfacial
Polymerization to Decrease Skin Irritation of High Permeability UV
Absorber Ethylhexyl Methoxycinnamate. Gels.

[22] Simeoni S., Scalia S., Tursilli R., Benson H. (2006). Influence of Cyclodextrin
Complexation on the in vitro Human Skin Penetration and Retention
of the Sunscreen Agent, Oxybenzone. J. Inclusion
Phenom. Macrocyclic Chem..

[23] Gulson B., Wong H., Korsch M., Gomez L., Casey P., McCall M., McCulloch M., Trotter J., Stauber J., Greenoak G. (2012). Comparison of Dermal
Absorption of Zinc from Different
Sunscreen Formulations and Differing UV Exposure Based on Stable Isotope
Tracing. Sci. Total Environ..

[24] Hameed M., Zameer A., Raja M. A. Z. (2024). A Comprehensive
Systematic Review:
Advancements in Skin Cancer Classification and Segmentation Using
the ISIC Dataset. Comput. Model. Eng. Sci..

[25] Kellermanni R. C. S., Camelo P. T. L. (2021). Uso de fotoprotetores na prevenção de
danos por exposição solar: conceitos, avaliação
histórica e recomendações. Scire Salutis.

[26] López-Hortas L., Torres M. D., Falqué E., Domínguez H. (2020). Organic UV
filter loaded nanocarriers with broad spectrum photoprotection. Nanocosmetics.

[27] Luppi B., Cerchiara T., Bigucci F., Basile R., Zecchi V. (2004). Polymeric
nanoparticles composed of fatty acids and polyvinylalcohol for topical
application of sunscreens. J. Pharm. Pharmacol..

[28] Alvarez-Roman X., Naik A., Kalia Y. N., Guy R. H., Fessi H. (2004). Enhancement
of Topical Delivery from Biodegradable Nanoparticles. Pharm. Res..

[29] Anumansirikul N., Wittayasuporn M., Klinubol P., Tachaprutinun A., Wanichwecharungruang S. P. (2008). UV-screening
chitosan nanocontainers:
increasing the photostability of encapsulated materials and controlled
release. Nanotechnology.

[30] Wu J., Liu W., Xue C., Zhou S., Lan F., Bi L., Xu H., Yang X., Zeng F.-D. (2009). Toxicity and penetration of TiO2
nanoparticles in hairless mice and porcine skin after subchronic dermal
exposure. Toxicol. Lett..

[31] Weiss-Angeli V., Bourgeois S., Pelletier J., Guterres S. S., Fessi H., Bolzinger M.-A. (2010). Development
of an original method to study drug release
from polymeric nanocapsules in the skin. J.
Pharm. Pharmacol..

[32] Senzui M., Tamura T., Miura K., Ikarashi Y., Watanabe Y., Fujii M. (2010). Study on penetration of titanium dioxide (TiO2) nanoparticles into
intact and damaged skin in vitro. J. Toxicol.
Sci..

[33] Vettor M., Bourgeois S., Fessi H., Pelletier J., Perugini P., Pavanetto F., Bolzinger M. A. (2010). Skin absorption
studies of octyl-methoxycinnamate loaded poly­(D,L-lactide) nanoparticles:
Estimation of the UV filter distribution and release behaviour in
skin layers. J. Microencapsulation.

[34] Siqueira N. M., Contri R. V., Paese K., Beck R. C. R., Pohlmann A. R., Guterres S. S. (2011). Innovative Sunscreen
Formulation Based on Benzophenone-3-Loaded
Chitosan-Coated Polymeric Nanocapsules. Skin
Pharmacol. Physiol..

[35] Marcato P. D., Caverzan J., Rossi-Bergmann B., Pinto E. F., Machado D., Silva R. A., Justo G. Z., Ferreira C. V., Durán N. (2011). Nanostructured
Polymer and Lipid Carriers for Sunscreen. Biological Effects and Skin
Permeation. J. Nanosci. Nanotechnol..

[36] Monteiro M. S. S. B., Ozzetti R. A., Vergnanini A. L., Brito-Gitirana L., Volpato N. M., Freitas Z. M. F., Ricci-Júnior E., Santos E. P. (2012). Evaluation of octyl p-methoxycinnamate included in
liposomes and cyclodextrins in anti-solar preparations: preparations,
characterizations and in vitro penetration studies. Int. J. Nanomed..

[37] Jirova, D. ; Kejlova, K. ; Pikal, P. ; Kasparova, L. ; Safarova, K. ; Kovarikova, L. ; Bendová, H. ; Zalabak, D. In Effect of TiO_2_ Nanoparticle Size on Possible Skin Penetration In Vitro; 14th Annual Congress of European Society for Alternatives to Animal Testing EUSAAT 2012 and the 17th European Congress on Alternatives to Animal Testing, Linz 2012, ATLA Alternatives to Laboratory Animals, 2012

[38] Hanno I., Anselmi C., Bouchemal K. (2012). Polyamide
Nanocapsules and Nano-emulsions
Containing Parsol MCX and Parsol 1789: In Vitro Release, Ex Vivo Skin
Penetration and Photo-Stability Studies. Pharm.
Res..

[39] Teixeira Z., Dreiss C. A., Lawrence M. J., Heenan R. K., Machado D., Justo G. Z., Guterres S. S., Durán N. (2012). Retinyl palmitate
polymeric nanocapsules as carriers of bioactives. J. Colloid Interface Sci..

[40] Miquel-Jeanjean C., Crepel F., Raufast V., Payre B., Datas L., Bessou-Touya S., Duplan H. (2012). Penetration Study of Formulated Nanosized
Titanium Dioxide in Models of Damaged and Sun-Irradiated Skin. Photochem. Photobiol..

[41] Puglia C., Damiani E., Offerta A., Rizza L., Tirendi G. G., Tarico M. S., Curreri S., Bonina F., Perrotta R. E. (2014). Evaluation
of nanostructured lipid carriers (NLC) and nanoemulsions as carriers
for UV-filters: Characterization, in vitro penetration and photostability
studies. Eur. J. Pharm. Sci..

[42] Crosera M., Prodi A., Mauro M., Pelin M., Florio C., Bellomo F., Adami G., Apostoli P., De Palma G., Bovenzi M., Campanini M., Filon F. L. (2015). Titanium Dioxide
Nanoparticle Penetration into the Skin and Effects on HaCaT Cells. Int. J. Environ. Res. Public Health.

[43] Xie G., Lu W., Lu D. (2015). Penetration
of titanium dioxide nanoparticles through
slightly damaged skin in vitro and in vivo. J. Appl. Biomater. Funct. Mater..

[44] Cerqueira-Coutinho C. S., Campos V. E. B., Rossi A. L., Veiga V. F., Holandino C., Maria Z. M. F., Ricci-Júnior E., Mansur C. R. E., Santos E. P., Santos-Oliveira R. (2016). Comparingin vivobiodistribution with radiolabeling
and Franz cell permeation assay to validate the efficacy of both methodologies
in the evaluation of nanoemulsions: a safety approach. Nanotechnology.

[45] Oliveira C. A. (2016). Safety
and Efficacy Evaluation of Gelatin-Based Nanoparticles Associated
with UV Filters. Colloids Surf. B Biointerfaces.

[46] Gilbert E., Roussel L., Serre C., Sandouk R., Salmon D., Kirilov P., Haftek M., Falson F., Pirot F. (2016). Percutaneous
absorption of benzophenone-3 loaded lipid nanoparticles and polymeric
nanocapsules: A comparative study. Int. J. Pharm..

[47] Joshi H., Hegde A. R., Shetty P. K., Gollavilli H., Managuli R. S., Kalthur G., Mutalik S. (2018). Sunscreen creams containing
naringenin nanoparticles: Formulation development and in vitro and
in vivo evaluations. Photodermatol., Photoimmunol.
Photomed..

[48] Andréo-Filho N., Bim A. V. K., Kaneko T. M., Kitice N. A., Haridass I. N., Abd E., Santos Lopes P., Thakur S. S., Parekh H. S., Roberts M. S., Grice J. E., Benson H. A. E., Leite-Silva V. R. (2018). Development
and Evaluation of Lipid Nanoparticles Containing Natural Botanical
Oil for Sun Protection: Characterization and in vitro and in vivo
Human Skin Permeation and Toxicity. Skin Pharmacol.
Physiol..

[49] Holmes A. M., Kempson I., Turnbull T., Paterson D., Roberts M. S. (2020). Penetration
of Zinc into Human Skin after Topical Application of Nano Zinc Oxide
Used in Commercial Sunscreen Formulations. ACS
Appl. Bio Mater..

[50] Khabir Z., Holmes A. M., Lai Y.-J., Liang L., Deva A., Polikarpov M. A., Roberts M. S., Zvyagin A. V. (2021). Human Epidermal
Zinc Concentrations after Topical Application of ZnO Nanoparticles
in Sunscreens. Int. J. Mol. Sci..

[51] Daneluti A. L. M., Guerra L. O., Velasco M. V. R., Matos J. R., Baby A. R., Kalia Y. N. (2021). Preclinical and
clinical studies to evaluate cutaneous
biodistribution, safety and efficacy of UV filters encapsulated in
mesoporous silica SBA-15. Eur. J. Pharm. Biopharm..

[52] Kaur (2022). ZnO Nanoparticles of *Rubia
cordifolia* Extract: Formulation and Optimization Using QbD. Molecules.

[53] Ma Q. (2023). Solid SiO_2_-Sealed Mesoporous Silica for Combined Organic and Inorganic
UV Filters. ACS Appl. Mater. Interfaces.

[54] Ghazwani M., Hani U., Alqarni M. H., Alam A. (2023). Development and Characterization
of Methyl-Anthranilate-Loaded Silver Nanoparticles: A Phytocosmetic
Sunscreen Gel for UV Protection. Pharmaceutics.

[55] de
Araújo M. M., Schneid A. C., Oliveira M. S., Mussi S. V., Miller N. F., Carvalho F. C., Junior E. A. B., Faro R., Azevedo H. (2024). NLC-Based Sunscreen Formulations with Optimized Proportion
of Encapsulated and Free Filters Exhibit Enhanced UVA and UVB Photoprotection. Pharmaceutics.

[56] Sousa I. P. (2024). Improved
Photostability and Skin Retention of Avobenzone Encapsulated in Nanostructured
Lipid Carriers. J. Cosmet. Sci..

[57] Tarantini A., Jamet-Anselme E., Lam S., Haute V., Suhard D., Valle N., Chamel-Mossuz V., Bouvier-Capely C., Phan G. (2024). Ex vivo skin diffusion and decontamination
studies of titanium dioxide
nanoparticles. Toxicol. In Vitro.

[58] Zhang J., Zhang S., Yan C., Bi J., Han X., Liu H. (2024). Tint-Adjustable Pickering Emulsion Sunscreen Based
on Polydopamine-Coated
Silica Nanoparticles. ACS Appl. Nano Mater..

[59] Gui H., Liu H., Cai Y., Nian J., Liu L., Song Y., Kye S., Zuo S., Yao C. (2025). Hyaluronic acid-grafted titanium
dioxide nanoparticles for moisture-retentive and non-cytotoxic sunscreen
creams. Int. J. Biol. Macromol..

[60] Shoaib
Khan H. M., Butt H., Sohail M., Rehman S., Ramzan N., Abbas Malik H. M. (2025). Pharmaceutical hybrid nanogel of
nano-flavonoid and zinc oxide for dermatological applications. J. Drug Delivery Sci. Technol..

[61] Li Z., Chen L., Qiu X. (2025). Green encapsulation
of avobenzone
in lignin microspheres: A promising approach for enhanced UV protection
and photostability. Ind. Crops Prod..

[62] Akombaetwa N., Ilangala A. B., Thom L., Memvanga P. B., Witika B. A., Buya A. B. (2023). Current Advances
in Lipid Nanosystems Intended for
Topical and Transdermal Drug Delivery Applications. Pharmaceutics.

[63] Kichou H., Bonnier F., Dancik Y., Bakar J., Michael-Jubeli R., Caritá A. C., Perse X., Soucé M., Rapetti L., Tfayli A., Chourpa I., Munnier E. (2023). Strat-M positioning
for skin permeation studies: A comparative study including EpiSkin
RHE, and human skin. Int. J. Pharm..

[64] Lucero M. J., Claro C., Casas M., Jiménez-Castellanos M. R. (2013). Drug diffusion
from disperse systems with a hydrophobically modified polysaccharide:
Enhancer vs Franz cells. Carbohydr. Polym..

[65] Bouwer F., Brits M., Viljoen J. M. (2025). Cracking the Skin Barrier: Models
and Methods Driving Dermal Drug Delivery. Pharmaceutics.

[66] Shetty P. K., Venuvanka V., Jagani H. V., Chetan G. H., Ligade V. S., Musmade P. B., Nayak U. Y., Reddy M. S., Kalthur G., Udupa N., Rao C. M., Mutalik S. (2015). Development and evaluation
of sunscreen creams containing morin-encapsulated nanoparticles for
enhanced UV radiation protection and antioxidant activity. Int. J. Nanomed..

[67] Rodrigues L. R., Jose J. (2020). Exploring the photo protective potential of solid lipid nanoparticle-based
sunscreen cream containing *Aloe vera*. Environ. Sci. Pollut. Res..

[68] Todo H. (2017). Transdermal
Permeation of Drugs in Various Animal Species. Pharmaceutics.

[69] Tomer S., Suh H., Zhou A. G., Yu B., Lewis J., Saltzman M., Girardi M. (2021). 504 Nanoparticle encapsulation
enhances stability and
efficacy of sunscreen actives. J. Invest. Dermatol..

[70] Tobler J. P., Rocha H. V. A. (2020). Bases regulatórias
para a avaliação
da segurança de medicamentos à base de nanotecnologia. Vigilância Sanitária Debate.

[71] Ouedraogo G., Alépée N., Tan B., Roper C. S. (2025). A call to action:
Advancing new approach methodologies (NAMs) in regulatory toxicology
through a unified framework for validation and acceptance. Regul. Toxicol. Pharmacol..

[72] Schmeisser S., Miccoli A., von Bergen M., Berggren E., Braeuning A., Busch W., Desaintes C., Gourmelon A., Grafström R. C., Harrill J., Hartung T., Herzler M., Kass G., Kleinstreuer N., Leist M., Luijten M., Marx-Stoelting P., Poetz O., Ravenzwaay B. V., Roggeband R. (2023). New approach methodologies in human regulatory
toxicology – Not if, but how and when!. Environ. Int..

[73] Romanhole R. C., Fava A. L. M., Tundisi L. L., Macedo L. M. d., Santos É. M. d., Ataide J. A., Mazzola P. G. (2020). Unplanned
absorption of sunscreen
ingredients: Impact of formulation and evaluation methods. Int. J. Pharm..

[74] Sitinjak F. Y., Aisyah N., Aulifa D. L., Budiman A. (2025). Advancements
in nanotechnology
for sunscreens: preparation, characterization, and mechanisms of UV
protection. OpenNano.

